# Diverse Heterologous Primary Infections Radically Alter Immunodominance Hierarchies and Clinical Outcomes Following H7N9 Influenza Challenge in Mice

**DOI:** 10.1371/journal.ppat.1004642

**Published:** 2015-02-10

**Authors:** Susu Duan, Victoria A. Meliopoulos, Jennifer L. McClaren, Xi-Zhi J. Guo, Catherine J. Sanders, Heather S. Smallwood, Richard J. Webby, Stacey L. Schultz-Cherry, Peter C. Doherty, Paul G. Thomas

**Affiliations:** 1 Department of Immunology, St. Jude Children’s Research Hospital, Memphis, Tennessee, United States of America; 2 Department of Infectious Diseases, St. Jude Children’s Research Hospital, Memphis, Tennessee, United States of America; University of Wisconsin-Madison, UNITED STATES

## Abstract

The recent emergence of a novel H7N9 influenza A virus (IAV) causing severe human infections in China raises concerns about a possible pandemic. The lack of pre-existing neutralizing antibodies in the broader population highlights the potential protective role of IAV-specific CD8+ cytotoxic T lymphocyte (CTL) memory specific for epitopes conserved between H7N9 and previously encountered IAVs. In the present study, the heterosubtypic immunity generated by prior H9N2 or H1N1 infections significantly, but variably, reduced morbidity and mortality, pulmonary virus load and time to clearance in mice challenged with the H7N9 virus. In all cases, the recall of established CTL memory was characterized by earlier, greater airway infiltration of effectors targeting the conserved or cross-reactive H7N9 IAV peptides; though, depending on the priming IAV, each case was accompanied by distinct CTL epitope immunodominance hierarchies for the prominent K^b^PB1_703_, D^b^PA_224_, and D^b^NP_366_ epitopes. While the presence of conserved, variable, or cross-reactive epitopes between the priming H9N2 and H1N1 and the challenge H7N9 IAVs clearly influenced any change in the immunodominance hierarchy, the changing patterns were not tied solely to epitope conservation. Furthermore, the total size of the IAV-specific memory CTL pool after priming was a better predictor of favorable outcomes than the extent of epitope conservation or secondary CTL expansion. Modifying the size of the memory CTL pool significantly altered its subsequent protective efficacy on disease severity or virus clearance, confirming the important role of heterologous priming. These findings establish that both the protective efficacy of heterosubtypic immunity and CTL immunodominance hierarchies are reflective of the immunological history of the host, a finding that has implications for understanding human CTL responses and the rational design of CTL-mediated vaccines.

## Author Summary

The emergence of human infections with a novel strain of avian-origin H7N9 virus in China raises a pandemic concern. The introduction of a new subtype in humans makes people at all ages susceptible due to the lack of population-wide neutralizing antibodies. However, cross-subtype protection from existing host immunity might provide important protection that can limit severe disease. Our study found that previous infection with non-H7N9 subtype viruses such as H9N2 viruses or H1N1 viruses could provide protection against lethal H7N9 challenge to varying degrees in mice. The virus-specific memory CD8+ T cells generated by the previous infection but targeting conserved or related portions of the internal proteins (epitopes) of the H7N9 viruses were selectively expanded and recruited at very early time points after H7N9 challenge, contributing to protective efficacy. The magnitude of the priming virus-generated memory CD8+ T cells was the best predictor of the protective efficacy of the heterosubtypic immunity against subsequent H7N9 challenge in these animals, when compared to the magnitude of the challenge response or the degree of epitope conservation between the priming and challenge strains. These results demonstrate the complexity of cross-reactive CD8+ T cell dynamics and suggest that significant protective immunity can be present even when few epitopes are conserved during heterosubtypic influenza infection.

## Introduction

Human infections with a novel, avian-origin H7N9 influenza A virus (IAV) were first seen in Southeast China in April 2013[1], re-emerged through the 2013 winter and spread to other regions [2]. Hospitalized cases were characterized by severe morbidity and, to date, around 30% mortality [1,3–5]. Although sustained person-to-person spread has not been observed, several amino acids in the virus hemagglutinin (HA) and polymerase acidic protein (PA) are known to be associated with efficient mammalian transmission and replication [6–9]. As H7N9 is a new serologic subtype for humans, there is little (or no) pre-existing neutralizing antibody in the population and all age groups are susceptible. Collectively, the severity of infection, the potential for transmission between humans, and the absence of neutralizing immunity raise serious concerns about a possible pandemic. This concern once again emphasizes the importance of cross-subtype protective mechanisms against influenza infection. There are indications [10–13] that prior infection with seasonal IAVs in humans can generate a measure of cross-reactive, or “heterosubtypic”, CD8+ cytotoxic T lymphocyte (CTL)-mediated immunity against further infection with other, serologically distinct seasonal or pandemic IAVs but, because of the limited case numbers to date, our understanding of how that plays out for H7N9 infection is rudimentary.

Human populations are repeatedly challenged by mutated variants of circulating IAVs (currently H1N1 and H3N2) that cause yearly “seasonal” epidemics, and by occasional, novel pandemic strains that are zoonotic reassortants. Estimates suggest that around 5–20% of people worldwide are infected annually with a seasonal IAV [14], while the global 2009 H1N1 infection rate was 20%–27% during the first pandemic year [15,16]. These viruses all carry immunogenic peptides that are broadly conserved [13,17]. Also, apart from natural exposure, live-attenuated IAV vaccines that grow in the upper respiratory tract can prime for a measure of heterosubtypic immunity [18,19]. In addition to the globally circulating human seasonal and pandemic strains, certain specific populations such as poultry workers may also have exposure to avian-origin IAVs. For example, the seroprevalance of H9-specific antibodies ranges from 3.5–15% in poultry workers from China and Vietnam [20–22] where H9N2 is prevalent in chicken populations. Most of these human H9N2 infections are clinically mild and underreported [23,24]. The majority of human H7N9 cases had a history of poultry contact in live poultry markets or farms [1,5,25], while direct contact with infected birds is characteristic of human H5N1 cases over the past decade [26], all indicating that poultry workers, and those who have close contact with live domestic birds have an enhanced likelihood of exposure to other avian IAVs.

In previous human [10–13,27] and animal studies [28–30], pre-existing heterosubtypic immunity, was unable to prevent secondary infection by other seasonal or pandemic IAVs, but clearly ameliorated morbidity and mortality, reduced virus load, and accelerated recovery in hosts, even for otherwise lethal doses of the highly pathogenic H5N1 virus [31,32]. Such protection have generally been attributed to the recall of cross-reactive CD8+ CTL effectors from memory immunity [30,33], though other mechanisms mediated by helper CD4+ T cells and by non-neutralizing antibodies may also be operating [33], either directly [34,35] or cooperatively with the CD8+ CTL response [36]. The virus-specific CTLs limit IAV replication by directly killing infected cells and by producing pro-inflammatory cytokines such as IFN-γ and TNF-α [37,38].

Clonotypic CTL T cell receptors (TCRs) recognize viral peptides (p) bound to cell-surface major histocompatibility complex (MHC) class I glycoproteins (pMHC or epitope). The majority of CTLs in any virus specific response target a limited number of epitopes from a potentially large pool [39,40], with the magnitudes of these diverse CTL responses sort into defined, reproducible, epitope immunodominace hierarchies. Of particular interest epidemiologically are those peptides that are shared by many different IAV subtypes [13,17]. With the H7N9 virus, for instance, two recent studies based on known human MHCI (HLA) presentation profiles identified substantial conservation of shared immunogenic peptides for the nucleoprotein (NP) and matrix-1 (M1) proteins [17],[41], suggesting that human H7N9 virus infection should recall cross-reactive CTL memory established by, for example, prior infection or vaccinations with seasonal H1N1 and H3N2 strains. Then, for Asian poultry workers, earlier exposure to H9N2 or other avian IAVs could also contribute to the memory CTL pool.

As we probe the possible protective efficacy of such pre-existing, heterotypic CTL memory to conserved epitopes, questions related to varying protective efficacy and also immunodominace hierarchies become of most interest. Such epitope-dependent differences in CTL response magnitude have been identified in humans though, because there are poorly understood interactive effects resulting from the expression of different MHCI alleles, questions related to mechanism are much more readily analyzed for inbred mouse strains. The IAV-specific immunodominance hierarchy has been particularly well characterized in the C57BL/6 (B6, H2K^b^D^b^) mice [42–45], using the mouse-adapted influenza strain A/ Puerto Rico/8/1934(H1N1) [PR/8(H1N1)] and the reverse-genetics virus A/Aichi/2/1968 X PR/8 [X31(H3N2)] that contains the six internal genes of the PR/8(H1N1) virus. During primary infection with the PR/8(H1N1) or X31(H3N2) viruses, the CTL response is directed predominantly against three epitopes: K^b^PB1_703_, D^b^PA_224_, and D^b^NP_366_, which together account for ~50% of the CD8+ T cells recovered from mouse airway during the peak adaptive response. The K^b^PB1_703_ and D^b^PA_224_ responses are co-dominant over D^b^NP_366_, and the persisting CTL memory pool maintains the same epitope hierarchy. Following secondary X31(H3N2) challenge of PR/8(H1N1)-primed mice, however, this CTL immunodominance profile is radically modified, with the D^b^NP_366_ response being much more predominant over K^b^PB1_703_ or D^b^PA_224_. However, even in mouse models, such CTL immunodominance hierarchies have not been well characterized for recent IAV field strains, either in primary nor heterosubtypic recall responses.

Here we investigate whether heterosubtypic CTL priming with different IAVs (including recent field isolates) can protect B6 mice against challenge with the novel H7N9 IAV. To maximize translational relevance, the experiments utilized two different H9N2 isolates, the 2009 pandemic H1N1 virus and the standard laboratory PR/8(H1N1) strain to probe the effect of varied priming regimes. The known dominant epitopes (K^b^PB1_703_, D^b^PA_224_ and D^b^NP_366_) have varying degrees of sequence identity for these different H9N2, H1N1 and H7N9 IAVs. We found that both clinical outcomes and the secondary CTL immunodominance hierarchies elicited by the same H7N9 challenge were dramatically influenced by the initial IAV exposure. The extent of epitope homology did not predict clinical outcome following the H7N9 challenge and that alone did not fully explain the observed changing immunodominace hierarchies either. These results demonstrate that the protective efficacy of CTL-mediated heterosubtypic immunity against lethal H7N9 infection is extremely sensitive to multiple features of the prior IAV exposure. This may go some way towards explaining varied human disease susceptibility profiles for individuals who have been exposed previously to one or more episodes of IAV infection.

## Results

### Priming and challenging infection regimes

**Figure 1:**
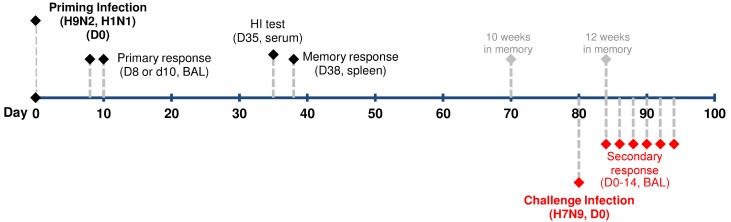
Experimental design for analyzing CTL-mediated heterosubtypic immunity against H7N9 virus infection. 8–10 week old female B6 mice were first primed intranasally with 10^4^ TCID_50_ of an H9N2 virus or 10^2^ TCID_50_ of an H1N1 virus. The virus-specific primary CTL responses in the bronchoalveolar lavage (BAL) were characterized on day (d) 8 and/or d10 post inoculation (p.i). Blood was collected for Hemagglutination inhibition (HI) assays on d35. The virus-specific memory CTLs in the spleen were characterized on d38. Between 10~12 weeks after the initial priming, the primed mice were intranasally challenged with an H7N9 virus and the H7N9 virus-specific-secondary CTL response in the BAL was characterized on various days between d0 to d14 after challenge infection.

The experimental design of sequential influenza virus infection (prime/challenge) in C57BL/6 (B6, H2K^b^D^b^) mice throughout these experiments is illustrated in Fig. 1. In every case, mice were infected intranasally (i.n.) with an IAV strain of a non-H7N9 subtype (H9N2 or H1N1) to generate immune memory. A criterion of a sublethal dose with the ability to induce seroconversion against homologous virus was used to choose the doses for the priming infection. 10^4^ TCID50 was previously determined to meet these criteria for both H9N2 viruses [46]. The same dose is 100% lethal for both H1N1 viruses, so a lower sublethal dose of 10^2^ TCID50 was chosen for those viruses. After ~10–12 weeks, primed (and naïve) mice were challenged i.n. with A/Anhui/01/2013(H7N9) [AH/01(H7N9)]. The IAV surface HA and NA glycoproteins of the priming viruses are serologically unrelated to those of the H7N9; the absence of antibody-mediated cross neutralization allows us to concentrate on other protective mechanisms, particularly the CTL response.

The four IAVs used for priming share different degrees of sequence identity with the H7N9 virus for the peptides that contribute to the three immunodominant epitopes (PB1_703_, PA_224_, and NP_366_) in B6 mice (Table 1). The A/Chicken/HongKong/TP38/2003(H9N2) [Ck/HK/TP38(H9N2)] strain is a direct chicken isolate, belonging to the G9 lineage [46] which is prevalent in Chinese chicken populations and is thought to have contributed the internal genes to the novel, reassortant H7N9 viruses; its three epitope-associated peptides are all identical to those expressed in AH/01(H7N9). The A/HongKong/33892/2009(H9N2) [HK/33892(H9N2)] virus is an avian-origin isolate from an infected human that has been recommended as a vaccine strain for the G1 lineage of H9N2 viruses and is representative of another lineage prevalent in Chinese chicken populations [46]; two of its epitope-associated peptides (PB1_703_, PA_224_) match those of AH/01(H7N9). The A/California/04/2009(H1N1) [CA/4(H1N1)] virus belongs to the 2009 pandemic H1N1 lineage and shares only one epitope-associated peptide (PB1_703_) with AH/01(H7N9), while the A/PR/8/34 PR/8(H1N1) laboratory strain has two epitope-associated peptides (PB1_703_, PA_224_) matching those of AH/01(H7N9).

**Table 1:** Sequence identity of CTL epitope-associated peptides in the studied viruses.

Virus strain	Epitopes in C57BL/6J Mouse^a^		
	K^b^PB1_703–711_	D^b^PA_224–233_	D^b^NP_366–374_
A/Anhui/1/2013 (H7N9)	SSYRRPVGI	SSLENFRAYV	ASNENMEAM
A/Chicken/Hong Kong/TP38/2003(H9N2)	∙ ∙ ∙ ∙ ∙ ∙ ∙ ∙ ∙	∙ ∙ ∙ ∙ ∙ ∙ ∙ ∙ ∙ ∙	∙ ∙ ∙ ∙ ∙ ∙ ∙ ∙ ∙
A/Hong Kong/33982/2009(H9N2)	∙ ∙ ∙ ∙ ∙ ∙ ∙ ∙ ∙	∙ ∙ ∙ ∙ ∙ ∙ ∙ ∙ ∙ ∙	∙ ∙ ∙ ∙ ∙ V ∙T ∙
A/Puerto Rico/3/1934(H1N1)	∙ ∙ ∙ ∙ ∙ ∙ ∙ ∙ ∙	∙ ∙ ∙ ∙ ∙ ∙ ∙ ∙ ∙ ∙	∙ ∙ ∙ ∙ ∙ ∙ ∙ T ∙
A/California/4/2009(H1N1)	∙ ∙ ∙ ∙ ∙ ∙ ∙ ∙ ∙	P ∙ ∙ ∙ ∙ ∙ ∙ ∙ ∙ ∙	∙ ∙ ∙ ∙ ∙ V ∙T ∙

^a^ ∙ represents an amino acid residue identical to that in the first row.

### Characteristics of primary infection with the H9N2 and H1N1 IAVs

**Figure 2:**
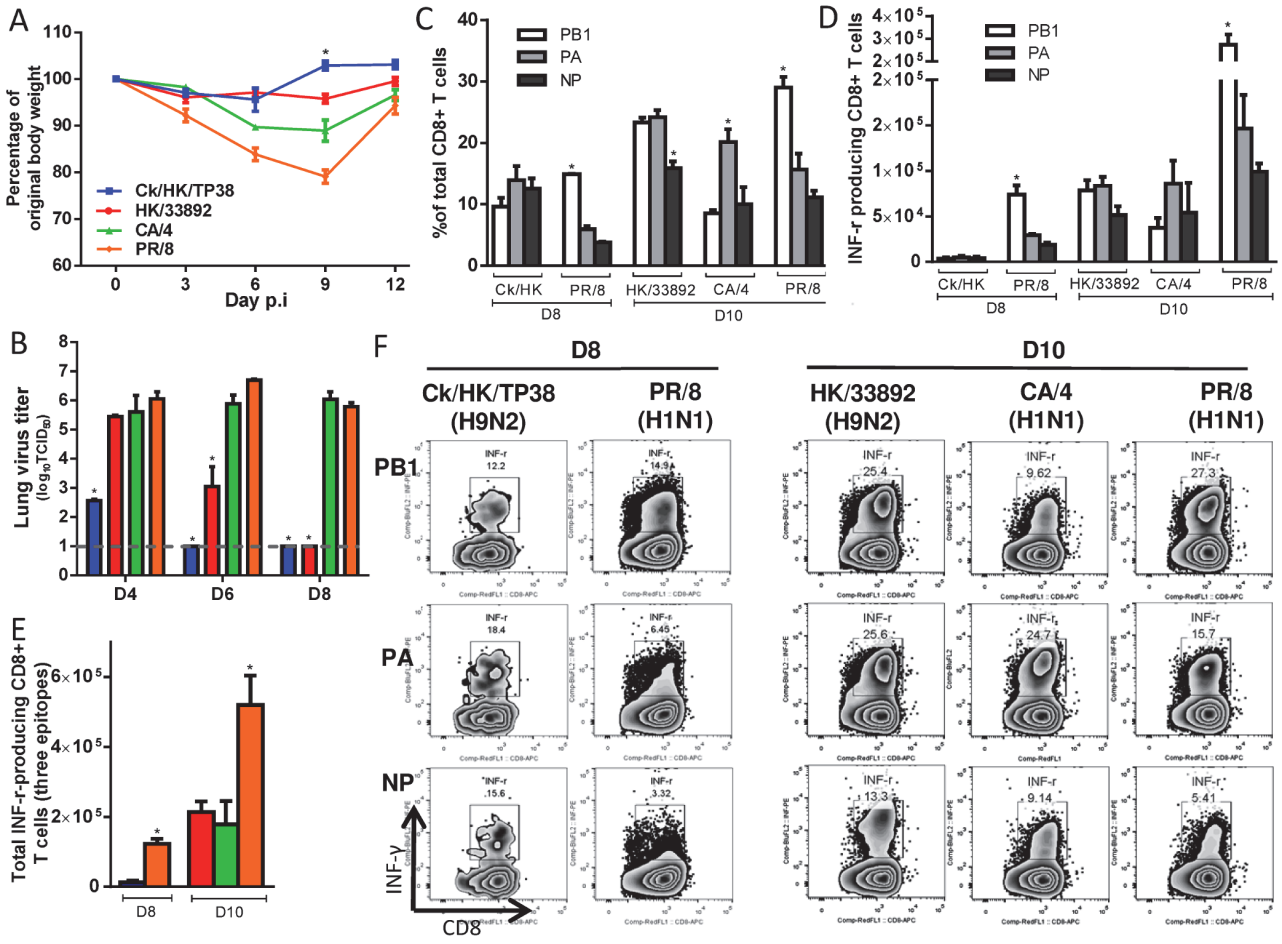
The disease course and primary CTL responses in naive mice after infection with one of two H9N2 or two H1N1 IAVs. The primary IAV infection was conducted as described in the legends to Fig. 1. (A) Body weight change and (B) virus replication kinetics in mice. (C) The proportion and (D) number of each epitope-specific CTL population, and (E) the total number of the three epitope-specific CTLs in the BAL on d8 or d10. (F) Representative IFN-γ ICS flow cytometry plots from CD8+ T cells in BAL samples. The PB1_703_, PA_224_, and NP_366_ peptide variants specific for each virus were used to stimulate the CTLs to produce IFN-γ for the ICS assay. The data sets represent mean ± SEM; * p<0.05 by Tukey’s test, comparing: (A, B) each virus *versus* every other virus at that time point, n = 10 per group; (C, D) the indicated epitope *versus* the other two epitopes in the virus, n = 4–5; (E) the indicated virus *versus* the other viruses.

The four priming viruses showed differing levels of pathogenicity (Fig. 2A) at the chosen sublethal dose (10^4^ TCID_50_ for the H9N2 and 10^2^ TCID_50_ for the H1N1 viruses) reflecting their different replication ability in mice (Fig. 2B). The two H9N2 viruses replicated relatively poorly and caused a minimal (or no) decline in body weight, while the CA/4(H1N1) and PR/8(H1N1) viruses replicated efficiently and induced moderately severe disease, with ~10% and 20% maximal weight loss, respectively. All infections led to seroconversion against the homologous strain, although significantly lower titers were found for the H9N2 versus the H1N1 IAVs (Table 2). Virus-specific CTLs were identified by specific peptide–stimulated IFN-γ production (ICS assay) in CD8+ T cells obtained by bronchoalveolar lavage (BAL) on day (d) 8 or d10 after infection (Fig. 2F), at the peak of the primary CTL response [43]. The peptide variant characteristic of each virus was used to induce IFN-γ production in CTLs. In each case, the CTL response to the three epitopes analyzed accounted for ~50% of the total CD8+ T cells infiltrating into the airways (Fig. 2C). In addition, the epitope immunodominance hierarchy varied for the different IAVs. The three epitopes were equally dominant in response to Ck/HK/TP38(H9N2) infection; the K^b^PB1_703_ and D^b^PA_224_ responses were equivalently dominant over D^b^NP_366_ for HK/33892(H9N2); the D^b^PA_224_ response was solely dominant following CA/4(H1N1) infection; K^b^PB1_703_ was dominant over D^b^PA_224_, followed by D^b^NP_366_, in PR/8(H1N1) infection, consistent with previous reports [43]. The total (for the 3 epitopes) magnitudes of the IAV-specific CTL responses induced by the four viruses also differed (Fig. 2D, E): PR/8 (H1N1) induced the largest numbers on d10, while Ck/HK/TP38(H9N2) caused a minimal response on d8 that was almost undetectable on d10, and the other two viruses were intermediate between these two extremes.

**Table 2:** Serologic testing of mice primed with different viruses.

Priming viruses	Serology Pre-challenge with Anhui/1(H7N9)						
	Young mice					Aged mice^**c**^	
	Against homologous virus		Against AH/1(H7N9)			Against homologous virus (HI)	Against AH/1(H7N9)(HI)
	HI^a^	MN^b^	HI	MN			
Ck/HK/TP38(H9N2)	6.67 ± 0.82	4 ± 0.63	< 2	< 2		4.66 ± 0.48*	< 2
HK/33892(H9N2)	7 ± 0.5	6.4 ± 0.49	< 2	< 2		6.37 ± 0.48*	< 2
PR/8 (H1N1)	10.25 ± 0.43	8.8 ± 0.4	< 2	< 2		NA	< 2
CA/4 (H1N1)	9.44 ± 0.68	8.6 ± 0.49	< 2	< 2		8.33 ± 0.7*	< 2
Naïve mice	< 2	< 2	< 2	< 2		<4	< 2

^a^ Hemagglutination inhibition assay (HI) or ^b^ Microneutralization assay (MN) was performed on serum samples collected on d35 p.i; Log_2_ of the HI or MN titers are expressed. Data represent mean ± SEM, n = 10–15/group.

^c^ Mice were primed at 16~18 months old.

* p<0.05, t test, aged *versus* HI titers in young group primed by the same virus.

**Figure 3:**
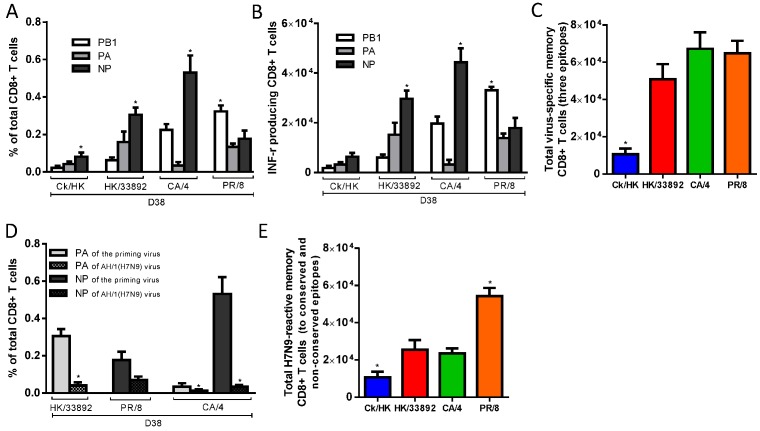
The prevalence of epitope-specific CTL memory cells after primary infection with the H9N2 and H1N1 IAVs. The primary IAV infection was conducted as described in the legends to Fig. 1. (A) The proportion and (B) number of epitope-specific memory CTL populations and (C) the total number of the three epitope-specific memory CTLs in spleen on d38; (D) The epitope-specific memory CTL populations generated by the priming infection that are cross-reactive with non-conserved epitopes in the H7N9 virus; (E) The total number of epitope-specific memory CTLs generated by primary infection targeting both the conserved and nonconserved epitopes in the H7N9 virus. (A-C) The PB1_703_, PA_224_, and NP_366_ peptide variants specific for each virus were used to stimulate memory CTLs to produce IFN-γ, except for (D) where cross-reactive variants were tested. The data sets represent mean ± SEM, n = 4–5 per group.* p<0.05 by Tukey’s test for (A,B, C, E) or by t test for (D), comparing: (A, B) the indicated epitope *versus* the other two epitopes; (C, E) the indicated virus *versus* the other three viruses; (D) the nonconserved *versus* the counterpart conserved epitope.

The resultant IAV-specific memory CTL counts were then determined by specific peptide–induced IFN-γ production for spleen populations taken on d38 (Fig. 3). While the K^b^PB1_703_ response remained dominant for PR/8(H1N1), the D^b^NP_366_ response persisted at greater levels for the other three IAVs (Fig. 3A,B). The total numbers of IAV-specific memory CTLs were highest for the two H1N1 viruses, lowest for Ck/HK/TP38(H9N2) and intermediate for HK/33892(H9N2) (Fig. 3B, C). In the mice primed with the two H9N2 viruses, the total lung resident CD8 T cells (S1 Fig.) were directly proportional to their sizes of the spleen memory CTL pool as well as the magnitude of the primary CTL response in the airway. In general, this reflected the magnitude of the primary CTL response measured in the airways (Fig. 2), supporting the view that the clonal burst at the acute stage of infection determines the size of the memory pool [43]. Together, the CTL and HI antibody response profiles indicated that Ck/HK/TP38(H9N2) is poorly immunogenic, HK/33892(H9N2) is intermediate and the two H1N1 viruses are strongly immunogenic, an effect clearly associated with their replication efficacy in mice.

We also assessed the level of epitope cross-reactivity (to H7N9) for the three priming viruses that carry differing PA_224_ and/or NP_366_ peptide sequences (Table 1). When the specific peptide variants of the respective priming viruses and AH/1(H7N9) were used to simulate IFN-γ production, only memory CTLs specific for the PR/8-D^b^NP_366_ epitope showed substantial cross-reactivity to the AH/1-D^b^NP_366_, at ~50% of the level found for the homologous peptide stimulation (Fig. 3D). The total number of memory CTLs reactive to the AH/1(H7N9) virus were defined as the epitope-specific cells targeting (and cross-reactive to) conserved and non-conserved peptides shared by the priming and challenge viruses. By this criterion, PR/8(H1N1) generated the highest level of CTL memory to AH/1(H7N9) virus, while Ck/HK/TP38(H9N2) was the least effective in this regard and the other two were intermediate (Fig. 3E).

In summary, the level of priming efficacy (measured by CTL numbers and HI antibody response) reflected the immunogenicity of the particular IAV which was, in turn, a likely consequence of the extent of virus replication in the lung. The replication ability of the priming IAVs were determined previously [46]: mammalian growth fitness was extremely low for Ck/HK/TP38(H9N2), intermediate for HK/33892(H9N2) and comparably high for both H1N1 viruses.

### Characteristics of primary infection with the H7N9 IAV

**Figure 4:**
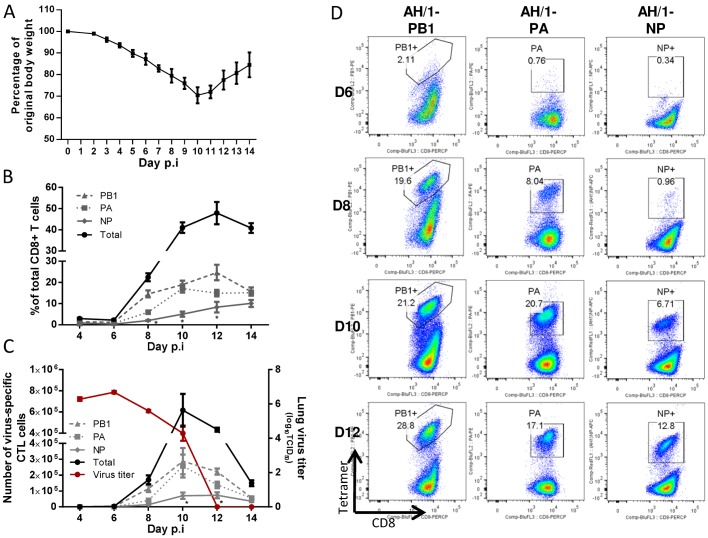
The disease course and primary CTL responses in naive mice after infection with the H7N9 IAV. Naïve mice were infected with 1 MLD50 (10^3.5^ TCID_50_) of the H7N9 virus. (A) Body weight change, (B) the proportion and (C, left Y axis) number of each epitope-specific CTL population in the BAL, and (C, right Y axis) the lung virus titer. Data sets represent mean ± SEM, n = 4–5 at each time point. * p<0.05, Tukey’s test, NP *versus* the other two epitopes. (D) Representative flow cytometry plots for each tetramer-specific CTL response in the BAL. The tetramers used were specific to the K^b^PB1_703_, D^b^PA_224_, and D^b^NP_366_ variants of the H7N9 virus.

Before assessing the consequences of the H7N9 challenge for these H1N1 and H9N2-primed mice, we first looked at the consequences of H7N9 infection in naïve B6 controls. The minimum lethal dose (MLD_50_) was determined as 10^3.5^ TCID_50_ in 8–10-week-old naïve, female mice. The mice experienced severe morbidity with approximately half succumbing to the infection. The surviving mice started to regain weight from about d11 (Fig. 4A), allowing evaluation of the disease course from infection to recovery. The virus replicated efficiently in the lung; the virus titer peaked on d6, decreased by d8 through d10, and was completely eliminated by d12 (Fig. 4C). Airway infiltration of virus-specific CTLs (detected by homologous tetramer staining) was not appreciable until d8 and peaked on d10, (Fig. 4B, C, D), corresponding to the kinetics of virus clearance (Fig. 4C). The CTL response to the three major epitopes accounted for ~50% of the CD8+ T cells infiltrating into the airway at the peak of the response (d10). The K^b^PB1_703_ response was detected early and K^b^PB1_703_ and D^b^PA_224_ responses were co-dominant over D^b^NP_366_ throughout (Fig. 4B, D).

### Outcomes of lethal challenge with H7N9 virus in the heterosubtypically-primed mice

**Figure 5:**
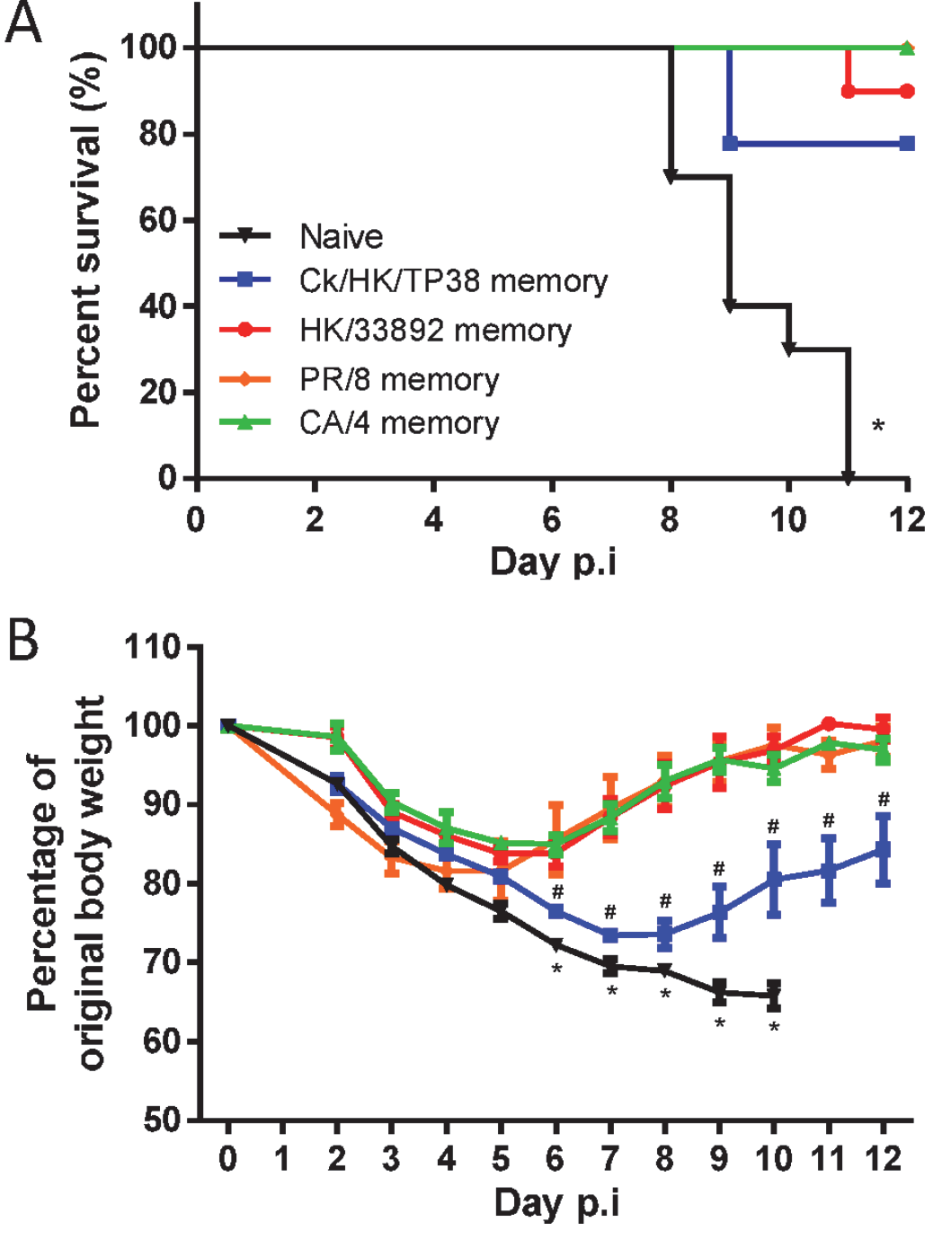
The disease course in naive or primed (with H1N1 or H9N2 IAVs 10–12 weeks previously) mice following challenge with the H7N9 virus. The mice were challenged with 10^4.5^ TCID_50_ H7N9 virus (10 MLD_50_). (A) The survival ratio and (B) weight loss during the disease course. Data represent mean ± SEM, n = 8–10/group from two independent experiments. (A) * p<0.05, log-rank test, naive *versus* other four primed groups. (B) * p<0.05, t test, naïve *versus* the every other four primed groups; # p<0.05, t test, Ck/HK/TP38 *versus* the every other three primed groups.

Next, we sought to determine whether the heterosubtypic immunity generated by these four serologically different (to H7N9) IAVs protects against lethal H7N9 challenge. Naïve or primed mice were challenged with 10^4.5^ TCID_50_ of H7N9 (10 MLD_50_ for naïve mice) and assessed for morbidity and mortality (Fig. 5). The naïve mice displayed severe morbidity, including rapid weight loss and other symptoms of clinical distress (including hunched back, ruffled fur and lethargy, data not shown), and all succumbed by d11. In contrast, those primed previously to the H9N2 or H1N1 IAVs were protected from mortality and morbidity to varying extents. All mice primed with the H1N1 viruses survived, compared with 90% survival for HK/33892(H9N2)-primed mice and 70% survival for Ck/HK/TP38(H9N2)-primed mice. Only the latter group showed severe and prolonged weight loss (~30% maximal); the other three groups all had short, mild disease courses (minimal weight loss, little observed clinical distress symptoms and rapid weight recovery). Clearly, heterosubtypic IAV immunity provides varying levels of protection against an otherwise lethal H7N9 infection.

### The H7N9 virus–specific CTL response in heterosubtypic immunity

We next examined the H7N9 virus-specific CTL populations infiltrating into the airway following the various priming regimes. Groups of primed and naive mice were challenged with 10^3.5^ TCID_50_ (1 MLD_50_ in naïve controls) of the H7N9 virus and CTL responses were evaluated as late as d10. At this challenge dose, all the primed mice survived with short and minimal weight loss, except for the Ck/HK/TP38(H9N2)-primed mice that had severe and prolonged weight loss (~25% maximal). Due to the constraints imposed by the need to conduct these experiments under BSL3 biosafety level, the evaluation of secondary CTL responses were conducted in different batches, with each batch including naïve controls for comparison which were consistently infected as described in Fig. 4.

**Figure 6:**
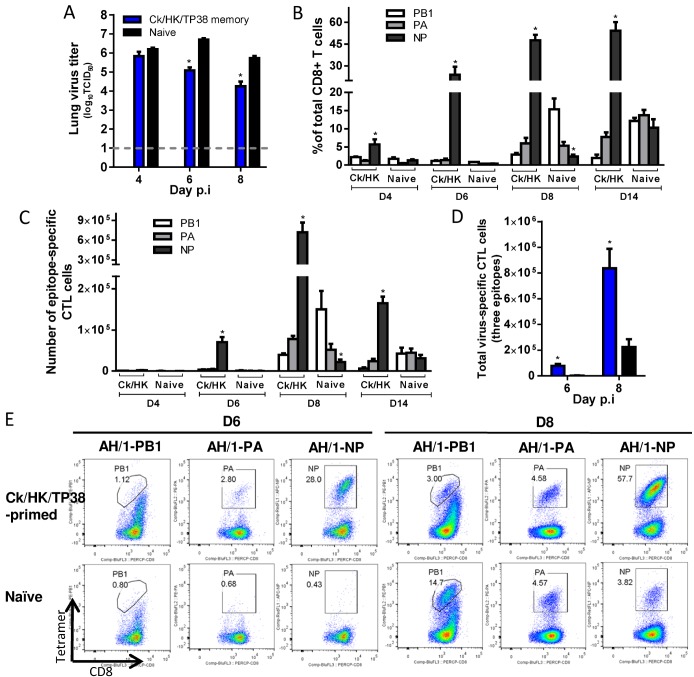
Comparing the primary and secondary CTL responses in naïve and Ck/HK/TP38(H9N2)-primed mice challenged with the H7N9 virus. The mice were challenged with 10^3.5^ TCID_50_ H7N9 virus (1 MLD_50_). (A) The virus titer in the lung and (B) proportion and (C) number of each epitope-specific CTL population and (D) the combined total number of three epitope-specific CTL populations in the BAL (data represent mean ± SEM, n = 4–5 at each time point). (A, D) * p<0.05, t test, primed *versus* naïve group. (B, C) * p<0.05, Tukey’s test, the indicated epitope *versus* the other two epitopes. (E) Representative flow cytometry plots for each tetramer-specific CTL population in the BAL. The tetramers used were specific to the epitope variants of the H7N9 virus.

**H7N9-specific CTL responses in Ck/HK/TP38(H9N2)-primed mice (Fig. 6)**. After AH/1(H7N9) virus challenge, for the Ck/HK/TP38(H9N2)-primed mice, the lung virus titers were ~1.5 log_10_TCID_50_ lower (versus naive controls) on d6 and d8 (Fig. 6A) H7N9 virus-specific CTLs infiltrated the airway from d4 and increased through d8 (Fig. 6B, C). Although all three epitopes are shared by the Ck/HK/TP38(H9N2) and AH/1(H7N9) viruses, and memory CTLs specific for all three could be recalled by the H7N9 challenge, the predominant recall response was to D^b^NP_366_ (Fig. 6B, C, E), accounting for ~50% of total infiltrating CD8+ T cells on d8 (~10–20-fold the D^b^PA_224_ or K^b^PB1_703_ responses). Both the proportion and the number of the dominant D^b^NP_366_ response in primed mice were significantly higher than those of the dominant K^b^PB1_703_ response in naïve mice at any time examined (Fig. 6B, C, E). The accumulated numbers for all three epitopes were ~33.1- and 3.7-fold that in the naïve mice on d6 and d8 respectively (Fig. 6D). Thus, the immune memory generated by Ck/HK/TP38(H9N2) protected against the secondary H7N9 challenge by reducing virus load, which was associated with the early and high infiltration of primed D^b^NP_366_-specific CTLs.

**Figure 7:**
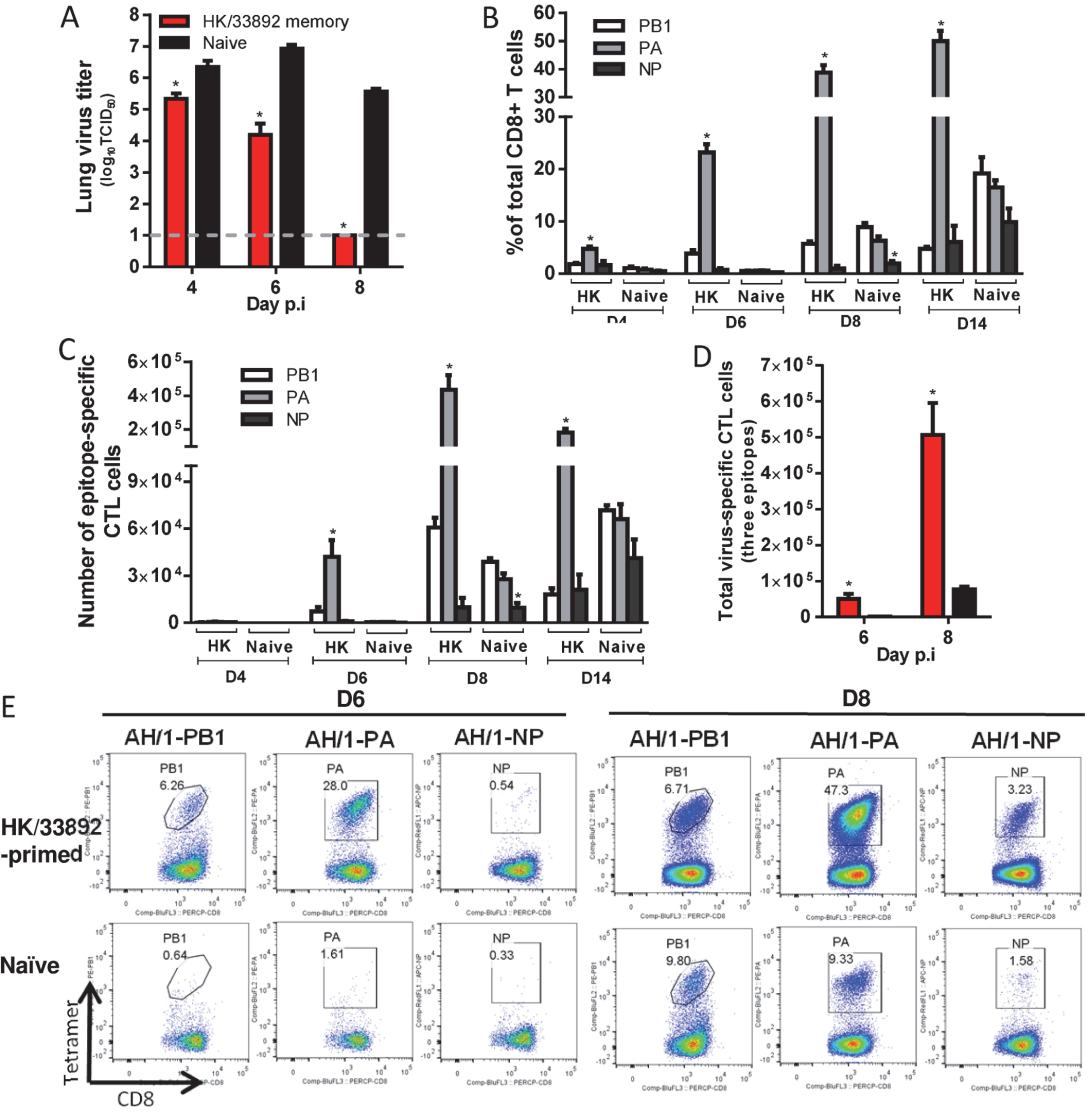
Comparing the primary and secondary CTL responses in naïve and HK/33892(H9N2)-primed mice challenged with the H7N9 virus. (A) The virus titer in the lung and (B) proportion and (C) number of each epitope-specific CTL population, and (D) the combined total number for the three epitope-specific CTL populations in the BAL. (E) Representative flow cytometry plots for each tetramer-specific CTL population in the BAL. Details of the data analysis and comparisons are same as shown in the legend to Fig. 6.

**H7N9-specific CTL responses in HK/33892(H9N2)-primed mice (Fig. 7)**. For the HK/33892(H9N2)-primed mice, the lungs had significantly lower virus titers than the naive mice on d4 and d6 (~1 and 2.7 log_10_TCID50 lower, respectively), and virus was completely cleared by d8 (Fig. 7A). Virus-specific CTLs began infiltrating the airway from d4 and continued increasing in numbers through d8 (Fig. 7B, C, E). Two epitopes (K^b^PB1_703_ and D^b^PA_224_) are shared between HK/33892(H9N2) and AH/1(H7N9), but the D^b^PA_224_-specific CTLs were preferentially recalled from memory to account for ~50% of the total infiltrating CD8+ T cells on d8 (~8–9-fold the K^b^PB1_703_ response) (Fig. 7B). Both the proportions and numbers of dominant D^b^PA_224_ response in primed mice were significantly higher than those of the dominant K^b^PB1_703_ response in naive mice at any time examined (Fig. 7B, C). The K^b^PB1_703_-specific recall response in primed mice occurred earlier and at a slightly higher level than the primary response in naïve mice. The total number of CTLs for the three epitopes was ~38.6- and ~5.7-fold that in the naïve mice on d6 and d8 respectively (Fig. 7D). Thus, the immune memory established by HK/33892(H9N2) protected against secondary H7N9 infection by expedited virus clearance mediated via early and high infiltration of primed D^b^PA_224_-specific CTLs.

**Figure 8:**
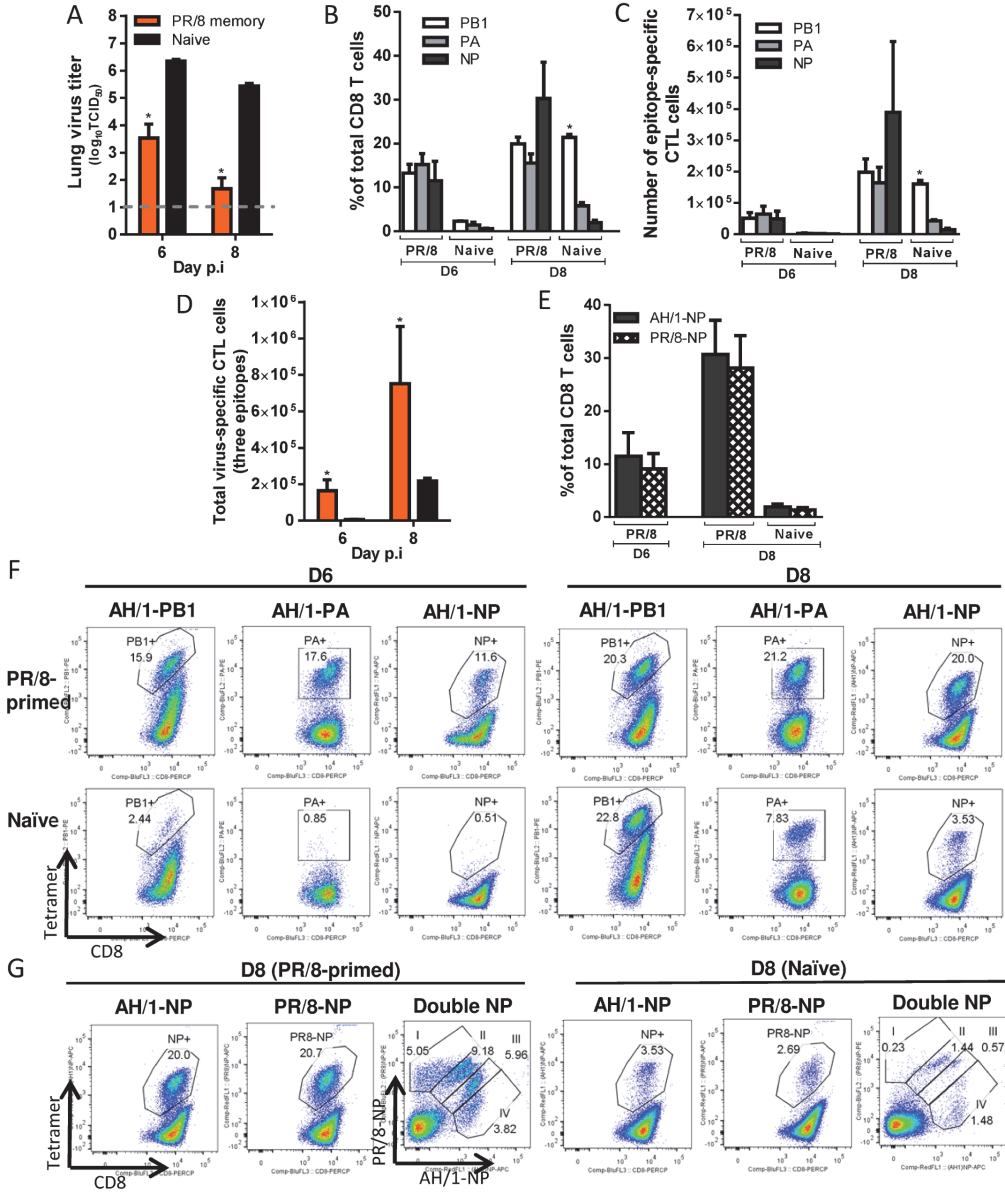
Comparing the primary and secondary CTL responses in naïve and PR/8(H1N1)-primed mice challenged with the H7N9 virus. (A) The virus titer in the lung and (B) proportion and (C) number of each epitope-specific CTL population, and (D) the combined total number for the three epitope-specific CTL populations in the BAL. (E) The proportions of CTL populations specific to AH/1-D^b^NP_366_ or PR/8-D^b^NP_366_ tetramers in the BAL. (F, G) Representative flow cytometry plots for each tetramer-specific CTL population in the BAL. Details of the data analysis and comparisons are same as shown in the legend to Fig. 6.

**H7N9-specific CTL responses in PR/8(H1N1)-primed mice (Fig. 8)**. For the PR/8 (H1N1)-primed mice, the lung virus titers were significantly lower (than naïve) on d6 and d8 (~2.8 and ~3.7 log_10_TCID50 lower, respectively) and half the mice had cleared the H7N9 challenge virus by d8 (Fig. 8A). As with HK/33892(H9N2), K^b^PB1_703_ and D^b^PA_224_ are shared (Table 1) by the PR/8(H1N1) and AH/1(H7N9) viruses, but the recall response to the two epitopes in PR/8(H1N1)-primed mice was entirely different from that described above for the HK/33892(H9N2)-primed mice (compare Figs. 7 & 8). Following PR/8(H1N1) priming, the recall response was equivalent on d6 and d8 to K^b^PB1_703_ and D^b^PA_224_, with each accounting for about 15–20% of total infiltrating CD8+ T cells (Fig. 8B, C, F). The NP_366_ sequence is not identical between the two viruses, differing at amino acid position 8 (T8A, Table 1), but the proportion of D^b^NP_366_-specific cells was equivalent to (or slightly higher than) those of recalled K^b^PB1_703_- and D^b^PA_224_-specific cells on d6 and d8 (Fig. 8B, C, F). Thus, all three epitopes became co-dominant in the PR/8(H1N1)-primed mice, distinct from the D^b^PA_224_-predominant response in HK/33892(H9N2)-primed mice. The total CTL responses to the three epitopes in the primed mice were significantly higher in proportion and number than those in the naive mice (Fig. 8C, D); the total counts were ~25.6 and ~3.5 times that in naïve mice on d6 and d8 (Fig. 8D).

The unexpectedly high proportion of AH/1-D^b^NP_366_–specific CTLs in PR/8(H1N1)-primed mice caused us to ask if, though the NP_366_ peptides in these two viruses are not identical, this reflected the cross-reactive recall of CTL memory (Fig. 3D). We thus examined the relative proportions of PR/8-D^b^NP_366_ and/or AH/1-D^b^NP_366_ –specific populations using tetramers specific for each epitope variant. Separate staining with each D^b^NP_366_ tetramer indicated that these two CTL sets were essentially equivalent in magnitude on d6 and d8 (Fig. 8E, G), while a further, double staining analysis showed four sets of the cells with varying levels of reactivity to the AH/1- or PR/8-D^b^NP_366_ tetramer binding (Fig. 8G). Double-stained cells accounted for the majority of the D^b^NP_366_ response following the AH/1(H7N9) challenge (Fig. 8G). Thus, the D^b^NP_366_ epitopes for these two viruses are cross-reactive, suggesting that a substantial proportion of PR/8-D^b^NP_366_-specific memory set was recalled by the H7N9 infection, reflecting the D^b^NP_366_ cross-reactivity found earlier for the memory response (Fig. 3D). Together, the memory immunity to the PR/8(H1N1) virus protects against H7N9 challenge by promoting rapid virus clearance mediated via the early and high infiltration of primed CTLs targeting (or cross-reactive) to all three epitopes of the H7N9 virus.

**Figure 9:**
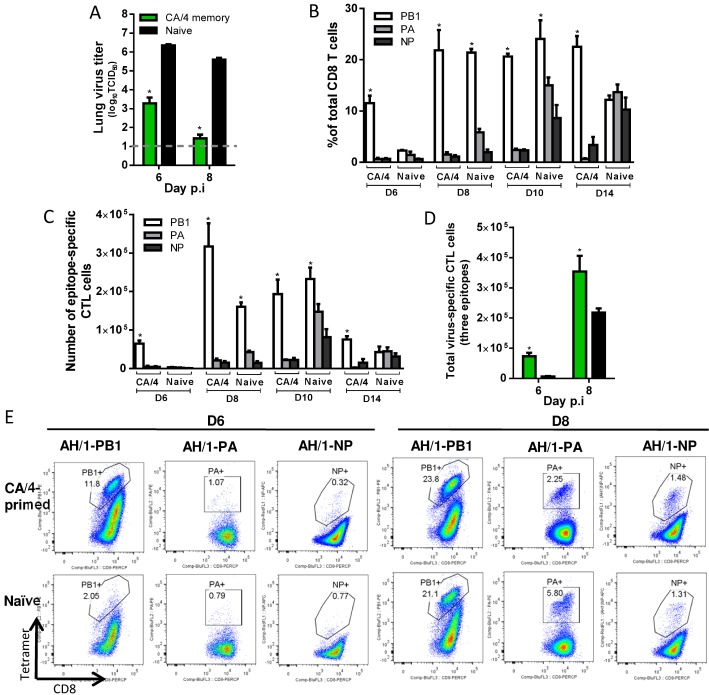
Comparing the primary and secondary CTL responses in naïve and CA/4(H1N1)-primed mice challenged with the H7N9 virus. (A) The virus titer in the lung and (B) proportion and (C) number of each epitope-specific CTL population, and (D) the combined total number for the three epitope-specific CTL populations in the BAL. (E) Representative flow cytometry plots for each tetramer-specific CTL population in the BAL. Details of the data analysis and comparisons are same as shown in the legend to Fig. 6.

**H7N9 virus-specific CTL responses in CA/4(H1N1)-primed mice (Fig. 9)**. After the H7N9 challenge, for the CA/4(H1N1)-primed mice, lung titers were significantly lower than those in the naïve mice on d6 and d8 (~3 and 4.2 log_10_TCID50 lower, respectively) (Fig. 9A). The only epitope shared by CA/4 (H1N1) and AH/1(H7N9) is K^b^PB1_703_; the early recall response to K^b^PB1_703_ on d6 accounted for ~12% of the total infiltrating CD8+ T cells (~7 times the K^b^PB1_703_ response in the naïve mice) (Fig. 9B, C, E). The proportion of K^b^PB1_703_-specific CTLs increased to, and maintained at, ~20% from d8 to d14, being ~10–20 times that of the D^b^PA_224_- or D^b^NP_366_ –specific populations in the primed mice (Fig. 9B, C, E). The numbers of K^b^PB1_703_ response in primed mice were significantly higher than those in naïve mice on d6 and d8. The total number of CTLs targeting the three epitopes was ~11.5 and ~1.6 times that in the naïve mice on d6 and d8 respectively (Fig. 9D). Thus, the immune memory established by CA/4(H1N1) priming protected against secondary H7N9 infection by expediting virus clearance mediated via early and high infiltration of primed K^b^PB1_703_-specific CTLs.

Together, first, our results show that prior exposure to serologically different IAVs can indeed provide a measure of protection against challenge with a virulent, novel influenza strain. Such protection is broadly associated with the early and high infiltration of primed H7N9 virus-specific CTLs into the airway. Second, distinct immunodominance hierarchies are found for the primary and memory CTL responses to the priming H1N1 and H9N2 IAVs, and for the primary and secondary responses to the H7N9 challenge (Table 3). The CTL epitope hierarchy following secondary H7N9 challenge depends in part on the extent of epitope sequence homology between the priming and challenging IAVs but other factors also contributed to the different recall hierarchies, as evidenced by the findings for HK/33892(H9N2)- and PR/8(H1N1)-priming cases (Fig. 7, 8) which have similar epitope conservation profiles. Third, the data presented above for each secondary CTL response suggested that any one of the D^b^NP_366_-specific, D^b^PA_224_-specific or K^b^PB1_703_-specific CTL sets are effectual to mediate virus clearance.

**Table 3:** Summary of virus-specific CTL epitope hierarchies.

Virus Name	Primary response (D8 or d10)	Memory response (D38)	Secondary response to H7N9 challenge (D70 after priming infection)	
			Matched epitopes (between 1^st^ and 2^nd^ viruses)	Secondary response (D8 after 2^nd^ infection)
AH/1(H7N9)	PB1 ≈ PA > NP	/	/	/
Ck/HK/TP38(H9N2)	PB1 ≈ PA ≈ NP	NP > PA ≈ PB1	PB1, PA, NP	NP >> PA > PB1
HK/33982(H9N2)	PB1 ≈ PA > NP	NP > PA ≈ PB1	PB1, PA	PA >> PB1 > NP
PR/8(H1N1)	PB1> PA > NP	PB1 > PA ≈ NP	PB1, PA	PB1 ≈ PA ≈ NP
CA/4(H1N1)	PA > PB1 ≈ NP	NP > PB1 > PA	PB1	PB1 >>PA ≈ NP

### Correlation between protective efficacy and heterosubtypic immunity

The heterostutypic immunity generated by the two H1N1 viruses was slightly more protective against the H7N9 challenge in terms of disease morbidity, mortality, and virus clearance than that generated by the HK/33892(H9N2), which was much greater than that generated by the Ck/HK/TP38(H9N2). The extent of epitope conservation could not explain this outcome as Ck/HK/TP38(H9N2) shares all three major epitopes with the H7N9 challenge. The observed differential protective efficacy prompted us to seek for correlations with other parameters of heterosubtypic immunity.

**Figure 10:**
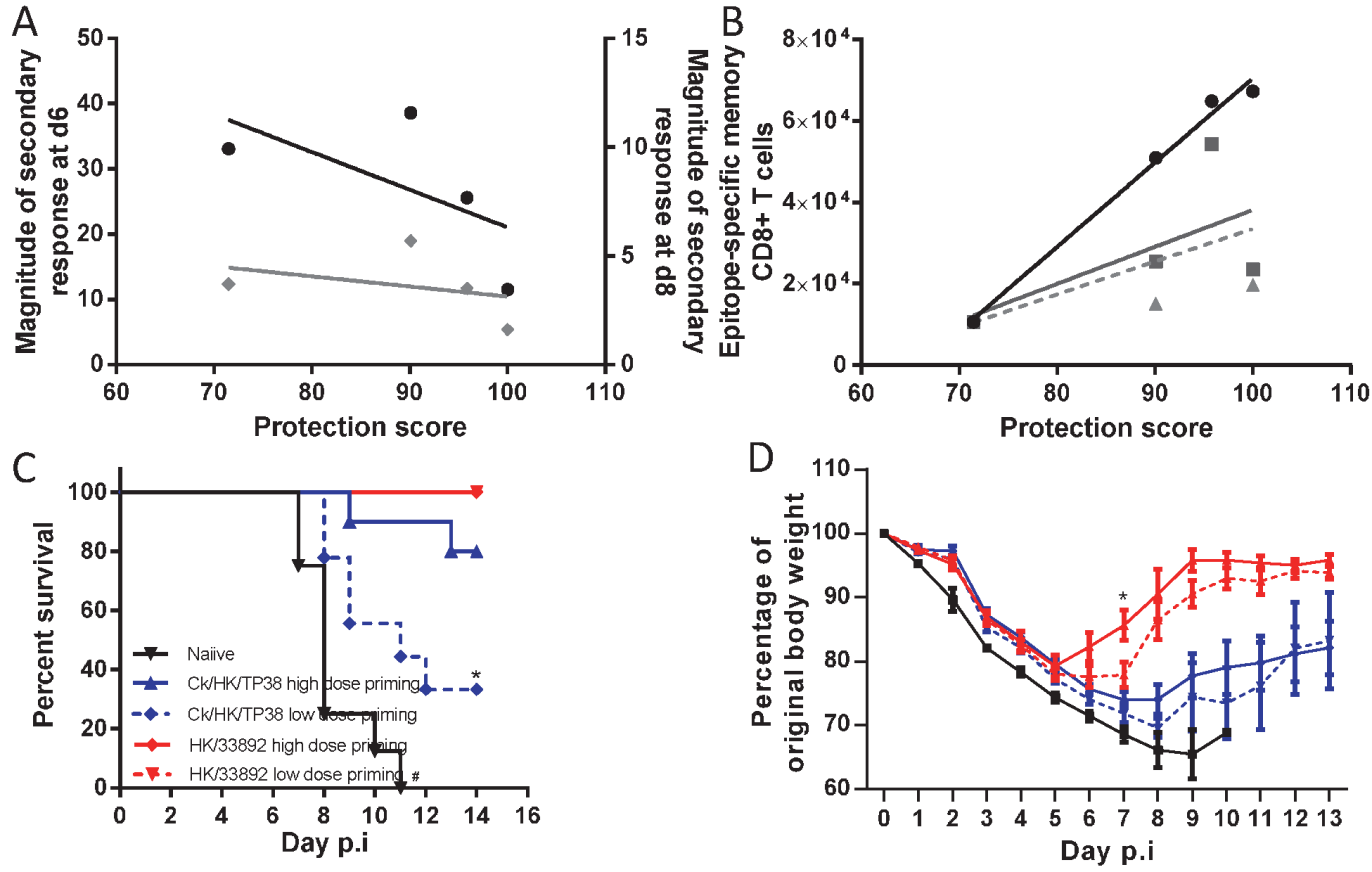
Correlation analysis of the protective efficacy and heterosubtypic immunity. Pearson correlation analysis of the protective efficacy with (A) the magnitude of the H7N9 virus-specific secondary CTL response in the airways on d6 (black line, left Y axis) and d8 (grey line, right Y axis), (B) the total number of priming-virus-specific memory CTL populations generated by the homologous virus (black line); the total number epitope-specific memory CTL populations targeting the conserved and non-conserved cross-reactive epitopes in the H7N9 virus (solid grey line); the number of epitope-specific memory CTL populations which were dominantly recalled during later during challenge infection (dashed grey line). The protective efficacy score was defined by a combination of survival ratio, body weight retention and virus clearance rate; the values of each parameter were normalized to the % maximum and the final score was the average of the three parameters. For (A), because the secondary infections were conducted in different batches, the fold-relationships for a given secondary CTL response in primed mice relative to the primary CTL response in the matched naïve mice were used to enable comparison between experiments. (C) The survival ratio and (D) weight loss of the mice primed with high or low dose of the H9N2 virus after they were challenged with 10^4.5^ TCID_50_ H7N9 viruses. Data represent mean ± SEM, n = 8–10/group, (C) * # p<0.05, log-rank test, * naïve versus other four primed groups, # Ck/HK/TP38 low dose *versus* high dose and naïve. (D) * p<0.05, t test, HK/33892 high dose *versus* low dose and naïve.

We examined the correlation between protective efficacy and 1) the magnitude of the H7N9 virus-specific CTL responses in the airway on d6 and d8 after secondary challenge (Fig. 10A); 2) the total counts for virus-specific memory CTLs generated by the priming infection; 3) the total number of epitope-specific memory CTLs reactive to the epitopes of the challenge H7N9 virus, and 4) the total number of the epitope-specific memory CTLs which later were predominantly recalled following the H7N9 challenge (Fig. 10B).

Protective efficacy was not correlated with the magnitude of the total, secondary, virus-specific CTL response in the airways on either d6 or d8 (R^2^ = 0.12 and 0.38 respectively). However, it was significantly correlated with the total size of the virus-specific memory CTL pool generated by the first virus infection (R^2^ = 0.99, p = 0.005), calculated by the total memory CTL counts for the three epitopes in the particular priming IAV which matched, to a greater or lesser extent, those in the challenging H7N9 virus (numbers in Fig. 3C). Therefore, we next asked if the total number of epitope-specific memory CTLs targeting identical epitopes, or reacting to the variants in the H7N9 (numbers in Fig. 3E), showed a comparable correlation. This parameter was not, however, well-correlated with protective efficacy (R^2^ = 0.39). Then, as these epitope-specific memory CTLs targeting those in the H7N9 virus were distinctly recalled following challenge, we further refined the analysis by focusing on the number of epitope-specific memory cells which were later dominantly recalled. Again, this parameter was not well-correlated with protection (R^2^ = 0.26). In the latter two correlation analyses, the CA/4(H1N1) case was the most discordant as it provided higher protection efficacy but generated lower numbers of the above defined epitope-specific memory cells. In conclusion, these analyses indicate that the best correlate of protection against the H7N9 challenge was the size of the priming virus-specific memory CTL pool, irrespective of the extent of epitope sequence homology.

To validate this observed correlation, we assessed whether modifying the size of the memory CTL pool can affect its subsequent protective efficacy. Our earlier results suggested that significantly different antigen doses during the primary infection generated different sized pools of memory CTLs (Fig. 2, 3), so we primed mice with a higher (10^5^ TCID50) or lower dose (10^3^ TCID50) of the H9N2 viruses. For each H9N2 virus, the higher dose priming generated a larger memory CTL pool than the lower dose priming; both doses of HK/33892 virus consistently generated a memory CTL size >5 times of that generated by same dose of Ck/TP/38 virus (S2 Fig.). Following challenge of H7N9 (Fig. 10C, D), all naïve mice rapidly succumbed by d11; all HK/33892-primed mice survived (both high and low dose-primed), but low dose-primed mice showed greater and longer weight loss than the high dose primed mice (~25% vs 20% maximal weight loss, 7 vs 5 days of weight loss). Mice primed with the high dose of Ck/HK/TP38 virus had 80% survival, in sharp contrast to 33% survival for the lower-dose primed mice and the survived mice from both dose groups experienced severe and prolonged weight loss when compared to the HK/33892 mice. We further modified the memory CTL pool generated by HK/33892 virus priming by using anti-CD8 antibodies to deplete the circulating virus-specific CTLs during and shortly after virus priming (S3 Fig.). The antibody-treated animals had a significantly reduced dominance of the D^b^PA_224_ response when compared with isotype control animals indicating the effect of the depletion, though the profile was still distinct from a naïve response, suggesting that the depletion was not complete (although tests of the peripheral blood after depletion showed >99% clearance of CD8 T cells). Still, the CD8-depleted animals had significantly higher and prolonged virus replication than the isotype control mice (S3C Fig.). In sum, different sizes of memory CTL pools generated during priming infections were directly associated with their protective efficacy against the challenge infection.

### Outcomes of secondary H7N9 infection in aged mice

Demographic studies show that older individuals (≥ 65 years), are at greater risk of severe H7N9 infection [1,3,5]. The reasonable certainty that the elderly have previously experienced influenza suggests that this population is very likely to have established heterosubtypic CTL memory. We thus investigated whether heterosubtypic immunity in aged mice protects against H7N9 infection. Female, 16–18 months old mice were first infected with an H9N2 or H1N1 IAV, as described above, and challenged two months later with the AH/1(H7N9) virus. The survival rate and disease course was compared with those for age-matched and young (8–10 week old) naive mice.

Infection with the H9N2 or H1N1 viruses remained sublethal in the aged, with the disease course being similar to that observed above (Fig. 2) for young animals, though the homologous HI titers were significantly lower (Table 2). These primed mice were then challenged with either of two doses of the AH/1(H7N9) IAV: 10^3.5^ TCID_50_ (1 MLD_50_ for 8~10-week-old naive female mice) and 10^4.5^ TCID_50_, in order to equalize infectivity to the ones used in the young mice described above (Figs. 5–9).

**Figure 11:**
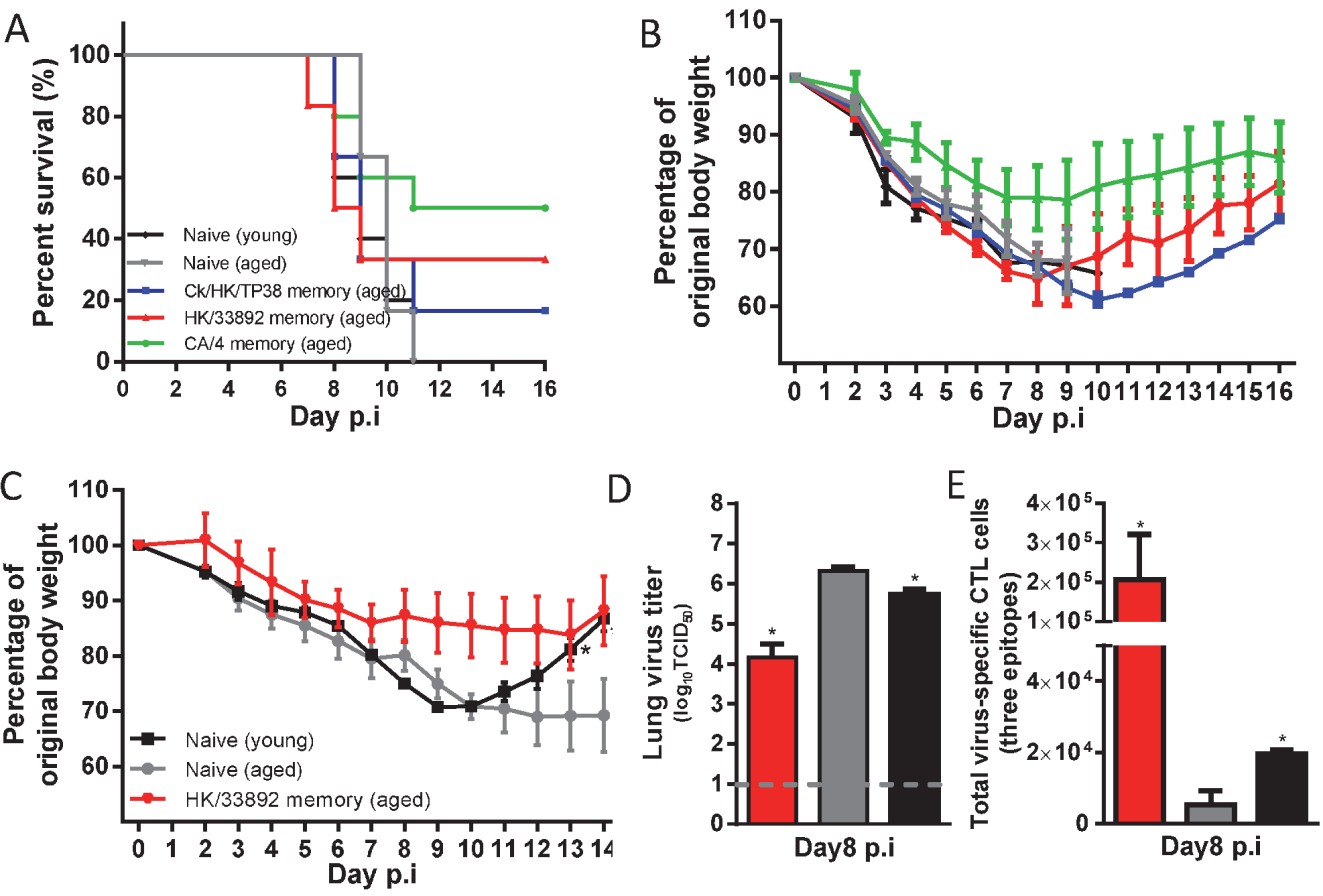
The disease course in young or aged naïve mice, or primed (with H9N2 or H1N1 virus) aged mice following H7N9 challenge. The aged females were between 16–18 months at priming and then were challenged about two months later or young (8–10 weeks). Aged matched or young (8–10 weeks) naïve female mice were used for comparisons. (A) The survival ratio and (B) body weight change of the mice after challenge with 10MLD50 of AH/1(H7N9) (data represent mean ± SEM, n = 5–6/group). (C) The body weight change, (D) virus titer in the lung, and (E) the total number of the three-H7N9 virus epitope-specific CTL populations in the BAL samples on d8 after challenge with 1MLD50 of AH/1(H7N9) virus (data represent mean± SEM, n = 3/group). (C, D, E) * p<0.05, t test, indicated group *versus* the naïve aged group.

Following challenge with 10^4.5^ TCID_50_ of the AH/1(H7N9) (Fig. 11A, B), both the aged and the young naïve mice succumbed to infection by d11, after rapid and severe weight loss. The aged mice primed with the different viruses were also severely affected (Fig. 11), with a survival rate of approximately 20%-50%, and all experiencing severe (~20–40% maximal) weight loss (Fig. 11A, B). Thus, following the H7N9 infection, there is (as in humans) an age-dependent effect on mice with enhanced mortality and morbidity (Fig. 5).

The 10^3.5^ TCID_50_ AH/1(H7N9) challenge caused young and aged naive mice to experience comparable weight loss and disease symptoms; approximately half succumbed and the surviving aged mice had a prolonged recovery course (Fig. 11C). Those primed with HK/33892(H9N2) all survived and showed less weight loss for this lower challenge dose (Fig. 11C). On d8, the aged naïve mice had slightly higher (~1 log_10_TCID_50_) lung virus titers than the young naïve mice, while the HK/33892(H9N2)-primed, aged mice had much lower (~4.2 log_10_TCID_50_) lung virus titers than the aged naïve mice (Fig. 11D). Young naive mice had slightly higher numbers of virus-specific CTLs in the airway than the aged naive mice (Fig. 11E). While priming with HK/33892(H9N2) led to the recall of significantly more virus-specific CTLs than those for either naïve group (Fig. 11E), the virus clearance was not as efficient as in the primed young mice (Fig. 8A). The epitope hierarchy pattern in the aged naive and primed mice was similar to that found earlier for young mice (data not shown).

Together, these results establish that, following primary H7N9 infection, advanced age is associated with smaller CTL responses, less efficient virus control in the lung, and a prolonged recovery course. As in young mice, heterosubtypic immunity provides protection against secondary H7N9 infection, but clinical outcomes (survival rate and weight retention) following the same challenge dose were substantially worse for aged mice, reflecting the demographics of human H7N9 infection.

## Discussion

Using a mouse model, we demonstrated that heterosubtypic immunity generated by a previous IAV infection provides varying degrees of protection against severe disease caused by an H7N9 virus challenge. Such protection was associated with the early and robust recall of IAV-specific CTLs targeting conserved, or cross-reactive, epitopes derived from the IAV internal proteins shared between different subtypes. We further found that dramatic and distinct shifts from the established CTL epitope immunodominance hierarchy (found following primary H7N9 infection) were characteristic of secondary H7N9 challenge in mice primed with different, serologically non-cross-reactive IAVs. Overall, we conclude that in heterosubtypic immunity, 1) the overall size of the priming-virus-specific memory CTL pool—regardless of the extent of epitope conservation between the priming and challenging viruses—is a positive correlate of protective efficacy against subsequent challenge; and 2) the changing epitope immunodominance hierarchies in secondary CTL responses depends only in part on epitope sequence homology between the two consecutively infecting IAVs.

Our study suggests that the magnitude of the total memory CTL pool generated by the priming virus is a key predictor of protective efficacy. This measure may serve as a proxy for the robustness and quality of the primary immune response that contributes independently to protective immunity, even when some dominant epitopes are mismatched. The size of the memory CTL pool has long been known to depend on the extent of antigen-driven clonal proliferation during the acute primary CTL response [47]. The observed direct association between the magnitude of the primary CTL response in the airways and the size of the memory CTL pool in the spleen later is in accord with this view, and holds for the four priming IAVs. Both the overall magnitude of the primary CTL response and the resultant memory pool correlates broadly with the growth fitness (in mice) of the various priming IAVs, which in turn manifests as protective efficacy against the H7N9 challenge. That is, more efficient IAV growth and higher immunogenicity of IAV in the primary infection generated a bigger memory pool, which gave more efficient protective immunity following subsequent heterosubtypic IAV challenge. It is possible that the size of the memory CTL pool could also reflect or influence very early, unmeasured effects in the local respiratory tract, such as the magnitude of the lung tissue resident memory T cell response, which has been found to have profound anti-viral effects other than direct cytotoxicity [48], or in the lymphoid tissue, such as memory cell activation and their homing to sites of infection in the lung. The size of memory CTL pool also could reflect the magnitude of other arms of the memory pool such as CD4+ T cells and B cells. Though the CTL response is likely to be the major contributor to heterosubtypic immunity, other immune mechanisms generated by the initial heterologous priming, such as helper CD4+ T cell effects and non-neutralizing antibodies, especially any mechanism that operates via cooperation with primed CTLs, could well play some part and are of interest for future studies.

We found no direct correlation between the magnitude of the secondary CTL responses in the airways and overall protective efficacy. If anything, there was a trend of inverse correlation for d8. It is likely that the observed magnitude of a given secondary CTL response in the airways reflects the feedback of the CTLs’ efficiency of virus clearance, with more efficient virus elimination resulting in less antigen availability and thus earlier, or faster, contraction of the expanded CTL pool. Connecting of the magnitudes of secondary CTL responses with their distinct epitope dominance patterns further suggests that the efficacy of particular CTL sets may vary depending on their epitope-specificity, therefore, leading to variation in protective efficacy. For example, the relatively low magnitude of the K^b^PB1_703_-dominant CTL response in CA/4(H1N1)-primed mice apparently provided a high level of protection, while the much larger, D^b^NP_366_-dominant CTL response in CkTP/38(H9N2)-primed mice provided relatively low protective efficacy. Several studies have found that D^b^NP_366_-specific effector CTLs showed general profiles of lower TNF-α and/or IFN-γ production and TCR/pMHCI avidity relative to those specific for the other two epitopes analyzed [49–51], and may thus be of lower functional quality. Also, though it has been suggested that D^b^PA_224_-specific CTLs is ineffectual [52], the present study supports the alternative view that they are highly protective. In general, the limited and somewhat controversial data on relative functional quality for different epitope-specific CTL effectors merits further analysis.

We found that epitope immunodominance hierarchies differed in each case we tested of the secondary CTL response, due to preferential recall of the particular epitope-specific memory CTL sets established by the heterosubtypic IAV priming. However, the epitope sequence homology alone in the two consecutive infecting viruses does not necessarily predict recall pattern. When only a single epitope was matched, not surprisingly, that epitope dominated the recall response (CA/4-priming case). In the case where all three epitopes were matched, the D^b^NP_366_ response was predominantly recalled (Ck/HK/TP38-priming case), consistent with the extensive prior findings for the PR/8 and X31 combination [43,52,53]. This has been explained by combinational factors including frequency of epitope-specific CTL precursors, cell surface antigen density [53], and differential antigen presentation [52]. However, in two cases where the same two (out of three) epitopes were matched, the recall responses were entirely different (HK/33892- and PR/8-priming cases). One important distinction was that the PR/8-D^b^NP_366_ was cross-reactive to the AH/1-D^b^NP_366_, while this is not the case for the HK/33892-D^b^NP_366_. These cross-reactive D^b^NP_366_-specifc CTLs may exert some “immunodominantion effect”, perhaps interfering with the activation of other memory cells by competing for or limiting access to epitopes on antigen presentation cells (APCs). Overall, our findings demonstrate the conditional nature of immunodominance, showing that it is not an intrinsic characteristic of either the host or the virus but the result of the complex interactions between them, opening questions for future mechanistic investigations.

One caveat of our immunodominance hierarchy analysis is that we only measured three dominant epitopes to represent the virus-specific CTL response and these three epitopes typically only represented ~50% of the total CTL response in the airways. Additional conservation or cross-reactivity among other minor epitopes which have not been identified yet could be occurring. Our previous study [45] analyzed seven additional predicted or previously characterized minor epitopes in a PR/8-priming/X31-chanllenge model and found that only a few epitopes, largely the three dominant epitopes analyzed in this study, were able to expand to significant levels in either the primary or secondary response, while the other minor epitopes maintained similarly low levels in both phases of responses. Only when the dominant epitopes were removed during primary infection was some compensatory expansion of a few minor epitopes observed. Thus, in light of the presence of conserved dominant epitopes in our study, we would hypothesize that any potential unknown individual minor epitopes are not likely to contribute substantially to the total response, though in combination they might represent most of the other 50% of uncharacterized CTL response in the airway. However, we cannot fully eliminate the possibility of a unique role for an unmeasured epitope now, and a better understanding of immune epitope generation and a full epitope search on each virus in each prime-challenge condition would be required to fully characterize the complete immunodomiance hierarchy profile.

It thus seems that, from our current understanding, we cannot consistently predict epitope dominance patterns following secondary infection, even when epitope identity between the priming and challenge IAVs is known. Whether epitope immunodominance patterns also operate similarly in human infections remains far from clear, mainly due to the uncertainty of any given individual’s infection history and the outbred nature (including HLA diversity) of the population. However, if our finding applies also to humans, it has substantial implications for the analysis of differential, HLA-related, CTL response profiles. Antigenically-drifted, seasonal IAVs and newly emerging pandemic strains often infect people who have, over the years, prior experience of one, or many, different IAV exposures. In such scenarios, even those who may be serologically naive for (in particular) a novel subtype may have CTL memory generated by those prior IAV infections. The likelihood is thus that the character and magnitude of any IAV-specific CTL response will be greatly influenced by the given individual’s immunological history, with there being considerable variation across the population. In this manner, epitope immunodominance shifts in heterosubtypic immunity would complicate CTL response analysis for individuals even of same HLA type, as the same epitope could elicit a primary response in a truly influenza-naïve individual, could be dominantly recalled, or less dominantly recalled or remain as a naïve epitope in the context of different heterosubtypic immunity. For example, the M1_58–66_ (GILGFVFTL)-specific CTL response tends to be prominent (and even exclusive) for some HLA-A*0201 subjects [54,55], but other studies have observed greater responses associated with the NP and PB1 proteins in others with this HLA type [56,57]. Variations in “background”, heterosubtypic immunity may account for such differences. As a consequence, extreme care should be taken in comparing the magnitude of epitope-specific CTL responses between different human subjects, even of the same HLA type. Both the combinatorial multiplex tetramer staining technique [58,59] and ICS assay following stimulation with a wide array of known peptides [13] provide approaches for looking more closely and precisely at these differential effects.

Our findings suggest that a prior infection of influenza A virus in humans could substantially change both the size and composition of the memory CTL pool, which could further substantially alter virus-specific CTL responses and disease severity of future secondary influenza A infections in the humans. The current limited numbers of cases and reported studies on H7N9 human infection have not been able to address the role of memory CTL-mediated protection yet. It is worth noting that currently all the laboratory-confirmed H7N9 cases are greatly skewed towards severe cases, most with underlying medical conditions and from elderly populations [1,3,4]. Recently, serology studies [60,61] demonstrated 6.3% -14.9% seroprevalence of anti-H7 antibodies among poultry workers in H7N9 endemic areas and increased anti-H7 antibody titers during the H7N9 outbreak, suggesting mild or subclinical infections might occur relatively commonly among poultry worker populations. It would be worthwhile in future studies on human infections to collect data on the patients’ previous influenza infection history, vaccination history, and seroconversion to other subtypes of prevalent avian and seasonal influenza viruses, and characterize the magnitude of virus-specific CTL responses in both severe and mild cases. Correlation analysis of these parameters with patient clinical outcomes may provide a useful means for disease prognosis of human H7N9 infection.

Our findings also have implications for evaluating the efficacy and rational design of CTL-directed immunogens, such as the live attenuated influenza vaccine (LAIV). Unlike the inactivated IAV vaccines, the LAIVs grow in the human upper respiratory tract, stimulating both humoral and cellular adaptive responses specific for the inducing strain [18,19,62]. Thus, the LAIVs have the potential to prime heterosubtypic CTL immunity in young children to provide some measure of protection against future, novel IAV infections. In adults who have experienced more than one episode of influenza, the LAIVs presumably serve as a relatively mild, secondary stimulus. For IAV vaccine design, the antigenic HA and NA proteins are evaluated for change annually but, for the LAIVs, a common donor “backbone” always provides the same internal proteins. Our study suggests that the protective efficacy of the LAIVs may be enhanced by increasing both the size and diversity of memory CTL populations targeting the typically dominant epitopes or cross-reactive epitopes though rational vaccine design.

We also assessed the effect of age on primary and secondary H7N9 infection in mice. People of all ages will be susceptible to a novel subtype like H7N9, with the elderly being at particularly high risk [1,3,5]. For humans [63–65], older individuals exhibit overall compromise in T cell functions such as proliferation, cytokine production, and cytotoxic mediator release. Mouse models have shown that aging reduces the host capacity to respond to novel pathogens [66,67], reflecting the production of fewer naïve T cells and diminished epitope-specific TCR diversity and CTL efficacy [68–70]. In the present study, aged, naïve mice, were more susceptible to primary H7N9 infection, with diminished CTL responses, less efficient virus control, and a prolonged disease course. Following H7N9 challenge, aged, primed mice were much less protected than young mice primed with the same virus. One study shows that late priming in aged mice can lead to effector and memory CTL populations with less TCR diversity and generate recall responses of lower magnitude, and these deficiencies can be partially overcome by early priming while the mice are still young [71]. Although memory responses were not directly compared for primed aged and young mice in our study, the aged subjects consistently showed lower HI titers, indicating reduced overall priming efficacy. Our results are consistent with the demographic profile that elderly humans are at higher risk for severe disease after H7N9 virus infection [3,5], supporting the view that health policy development and planning in the face of any IAV challenge needs to give this vulnerable group particular attention.

In summary, this study demonstrates 1) that the total size of the virus-specific memory CTL pool generated by a priming IAV is a key predictor of the protective efficacy of heterosubtypic immunity and; 2) that changing epitope immunodominance hierarchies characterize secondary CTL responses and are highly sensitive to IAV infection history. The findings presented here suggest new avenues for further investigation of the mechanisms modulating immunodominance and the functional quality of differential CTL effectors in heterosubtypic immunity. They also have implications for future studies of virus-specific CTL responses in humans and for the rational design of CTL-directed vaccines and immunotherapies.

## Materials and Methods

### Ethics statement

All animal studies were approved by the St Jude Children's Research Hospital Animal Care and Use Committee (Protocol number: 098), following the guidelines established by the Institute of Laboratory Animal Resources, approved by the Governing Board of the U.S. National Research Council.

### Mice

6–8 week-old C57BL/6 female and male mice were purchased from Jackson Laboratories (Bar Harbor, ME) and were acclimated to the St. Jude facility for two weeks prior to any experiment. Aged mice had been purchased from Jackson Laboratories at 6–8 week age and housed in an ABSL1 laboratory for 16–18 months. Inoculation with all viruses except for H7N9 viruses was conducted in an ABSL2+ facility; H7N9 virus inoculation was conducted in an ABSL3+ facility. Inoculated animals were assessed daily. Those with severe morbidity (greater than 30% weight loss plus severe clinical impairment) were humanely euthanized according to our approved protocol.

### Viruses and infections

The H9N2 and H7N9 viruses were provided by the World Health Organization Global Influenza Surveillance and Response System. All virus stocks were propagated in embryonated chicken eggs, and virus infectivity was measured by 50% tissue culture infectious dose (TCID_50_) in MDCK cells. The PR/8(H1N1) virus is a mouse adapted strain. Mice were chemically restrained with 2,2,2-tribromoethanol (Avertin) for intranasal delivery of 30 μl of virus diluted at the infectivity indicated in the Results section. After virus challenge, illness was monitored by daily weighing and assessment of the clinical distress symptoms such as ruffled fur, hunched back, lethargy etc.

### Synthetic peptides and tetramers

All peptides were synthesized by the Hartwell Center at St. Jude. Peptides corresponding to influenza CD8+ T cell epitopes NP_366–374_ variants and PA_224–233_ variants are all H-2Db–restricted. PB_1703-711_ is H-2Kb–restricted. Class I MHC tetramers were constructed by combining H-2Db or H-2Kb with the immunogenic peptides. Cells in BAL were stained with tetramers as described previously[45]. Briefly, cells were stained with allophycocyanin (APC)- or phycoerythrin (PE)-conjugated tetramers for 1 hour at room temperature. After washing, cells were stained with fluorescence-labeled antibodies to CD8α for 0.5 hour on ice. Cell samples were analyzed on a Calibur II flow cytometer (BD Biosciences).

### Intracellular cytokine staining and flow cytometry

After virus challenge, mice were euthanized at the described time and the BAL and mesenteric lymph nodes (MLN) were collected for analysis of primary and recall response; spleens were harvested for analysis of memory response. MLN and spleens were manually disrupted by grinding, and splenic red blood cells were lysed. Splenocytes were enriched for CD8+ cells by depleting CD4+ and B220+ cells. For intracellular cytokine staining (ICS), processed cell suspensions from BALF or spleen were stimulated with 1 μM of each virus-specific peptide variant in the presence of brefeldin A during culture in 96-well round-bottom plates for 5 hours at 37°C in 200 mcl RPMI containing 10% FCS. After in vitro stimulation, the cells were washed, fixed, and permeabilized according to the manufacturer's protocol (BD PharMingen Cytofix/Cytoperm kit). Cells were stained with anti-IFN-γ (clone XMG1.2) and anti-TNF (clone MP6-XT22) antibodies. Samples were analyzed on a Calibur II flow cytometer (BD Biosciences) and data were analyzed using FlowJo software (Tree Star, San Carlos, CA).

### Statistical analysis

Survival rates were compared using the log-rank test. The responses to the three epitopes were compared by one-way analysis of variance (ANOVA) and subsequent Tukey’s multiple comparisons. All other statistical comparisons used unpaired two sided t-tests, in Prism5 software. Data are presented as the mean ± SEM. The level of significance was determined as *P*<0.05.

## Supporting Information

S1 Fig Lung resident CD8 T cells at d35 after priming infection. Lung-resident CD8 T cells were detected using in vivo labeling. Anti-CD45 antibodies were i.v injected into mice before lung harvest. (A) Lung resident CD8 T cells were differentiated as negative for CD45, from blood-carrying circulating CD8 T cells which were positive for CD45 (right panel). Tetramer staining on blood-carrying (middle panel) or lung resident CD8 T cells (left panel). (B) Comparison of total lung resident CD8 T cells in the H9N2 virus-primed mice at d35 p.i. Data sets represent mean ± SEM, n = 5. * p<0.05, t test, Ck/HK/TP38 *versus* HK/33892.
(TIF)

S2 Fig Priming mice with different doses of the H9N2 viruses. (A) Weight loss in mice which were infected with a high (10^5^ TCID_50_) or a low dose (10^3^ TCID_50_) of the indicated H9N2 virus. (B) The total number of the three epitope-specific memory CTLs in spleen on d35 p.i generated by the H9N2 virus infection. Data sets represent mean ± SEM, (A) n = 15, (B), n = 5 per group.
(TIF)

S3 Fig The secondary response in HK/33892(H9N2)-primed mice after anti-CD8 antibody treatment. (A, B) Representative flow cytometry plots show the CD8 T cell population in blood samples after the HK33892(H9N2)-primed mice were administrated anti-CD8 antibody (Abs, left panel) or an IgG isotype control antibody (IgG, right panel) at d14 (A) and d35 p.i (B). The antibodies were injected into mice intraperitoneally at two days prior to priming infection and were further injected every three days for 2 weeks after the priming infection. They were then rested for at least a month prior to challenge with the H7N9 virus. (C)The virus titer in the lung and (D) the number of each epitope-specific CTL population (E) the combined total number of three epitope-specific CTL populations in the BALF after the antibody-treated mice were challenged with 10^4.5^ TCID50 H7N9 virus. The data sets represent mean ± SEM, n = 4–5 per group.* p<0.05, Tukey’s test, the indicated group *versus* the other two groups.
(TIF)

S4 Fig Secondary CTL response in aged mice primed with HK/33892(H9N2) virus. The aged female mice were between 16–18 months of age at priming and were challenged about two months after priming; age matched or young (8–10 weeks) naïve female mice were used for comparisons. The proportion (A) and number (B) of each epitope-specific CTL population in the BAL sample at d8 p.i. Data sets represent mean ± SEM, n = 3 per group. * *p<*0.05, Tukey’s test, the indicated epitope *versus* the other two epitopes.
(TIF)
