# Supplementary figures and images for: Diverse Heterologous Primary Infections Radically Alter Immunodominance Hierarchies and Clinical Outcomes Following H7N9 Influenza Challenge in Mice
Source: PLoS Pathog. 2015 Feb 10;11(2):e1004642. doi: 10.1371/journal.ppat.1004642 (PMC4335497; doi:10.1371/journal.ppat.1004642)

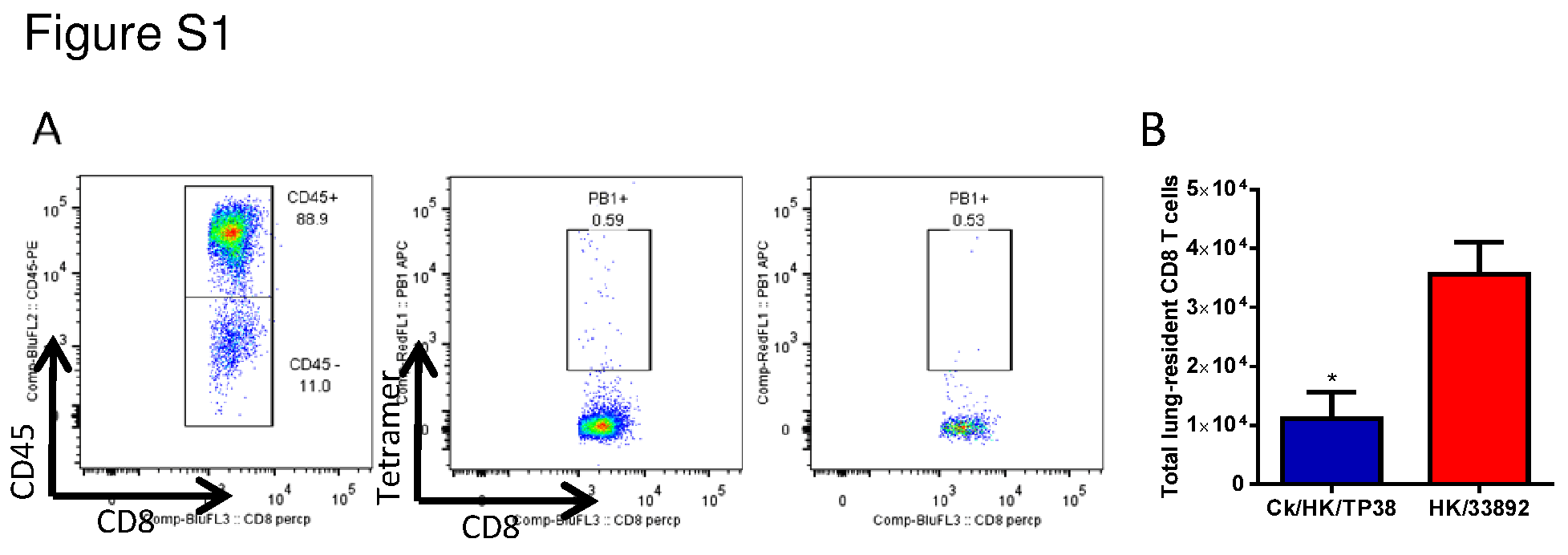

Supplement: S1 Fig — Lung-resident CD8 T cells were detected using in vivo labeling. Anti-CD45 antibodies were i.v injected into mice before lung harvest. (A) Lung resident CD8 T cells were differentiated as negative for CD45, from blood-carrying circulating CD8 T cells which were positive for CD45 (right panel). Tetramer staining on blood-carrying (middle panel) or lung resident CD8 T cells (left panel). (B) Comparison of total lung resident CD8 T cells in the H9N2 virus-primed mice at d35 p.i. Data sets represent mean ± SEM, n = 5. * p<0.05, t test, Ck/HK/TP38 versus HK/33892. (TIF) [file ppat.1004642.s001.tif]

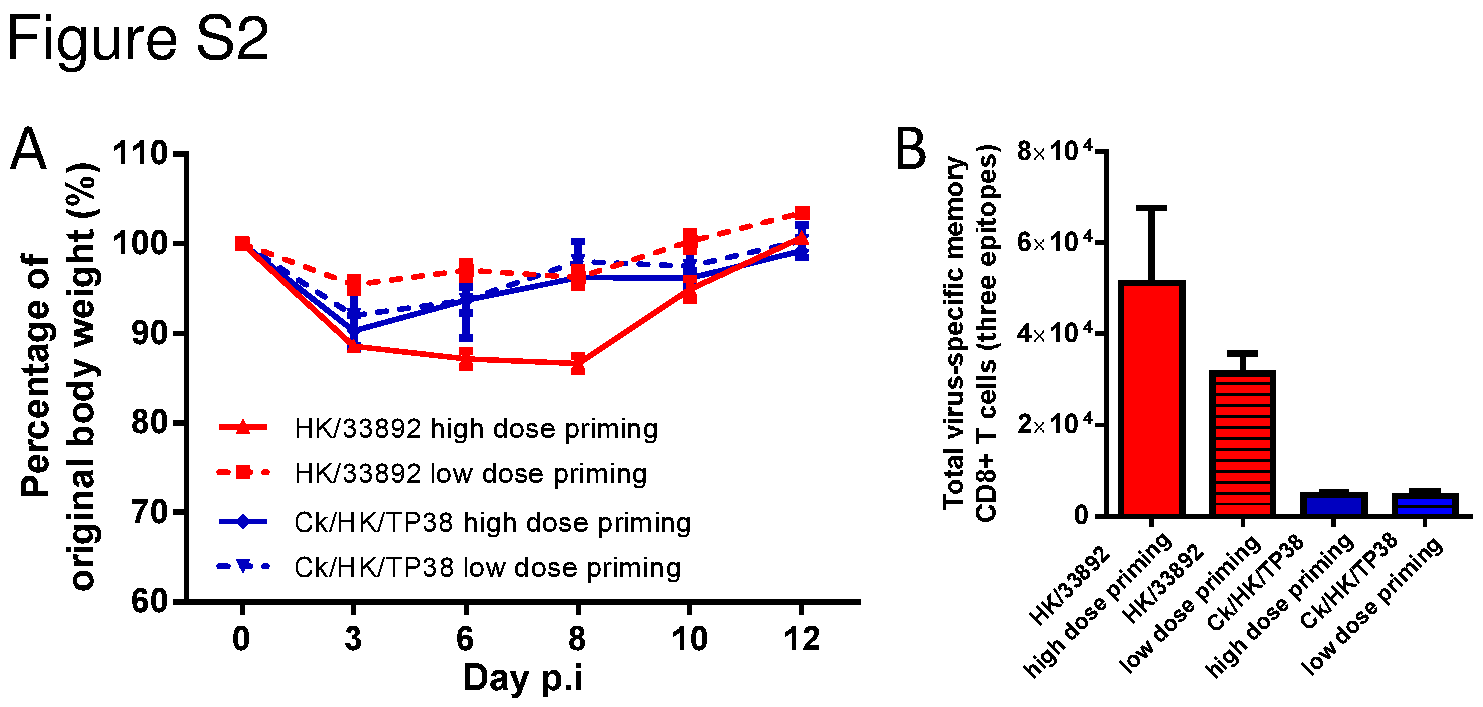

Supplement: S2 Fig — (A) Weight loss in mice which were infected with a high (105 TCID50) or a low dose (103 TCID50) of the indicated H9N2 virus. (B) The total number of the three epitope-specific memory CTLs in spleen on d35 p.i generated by the H9N2 virus infection. Data sets represent mean ± SEM, (A) n = 15, (B), n = 5 per group. (TIF) [file ppat.1004642.s002.tif]

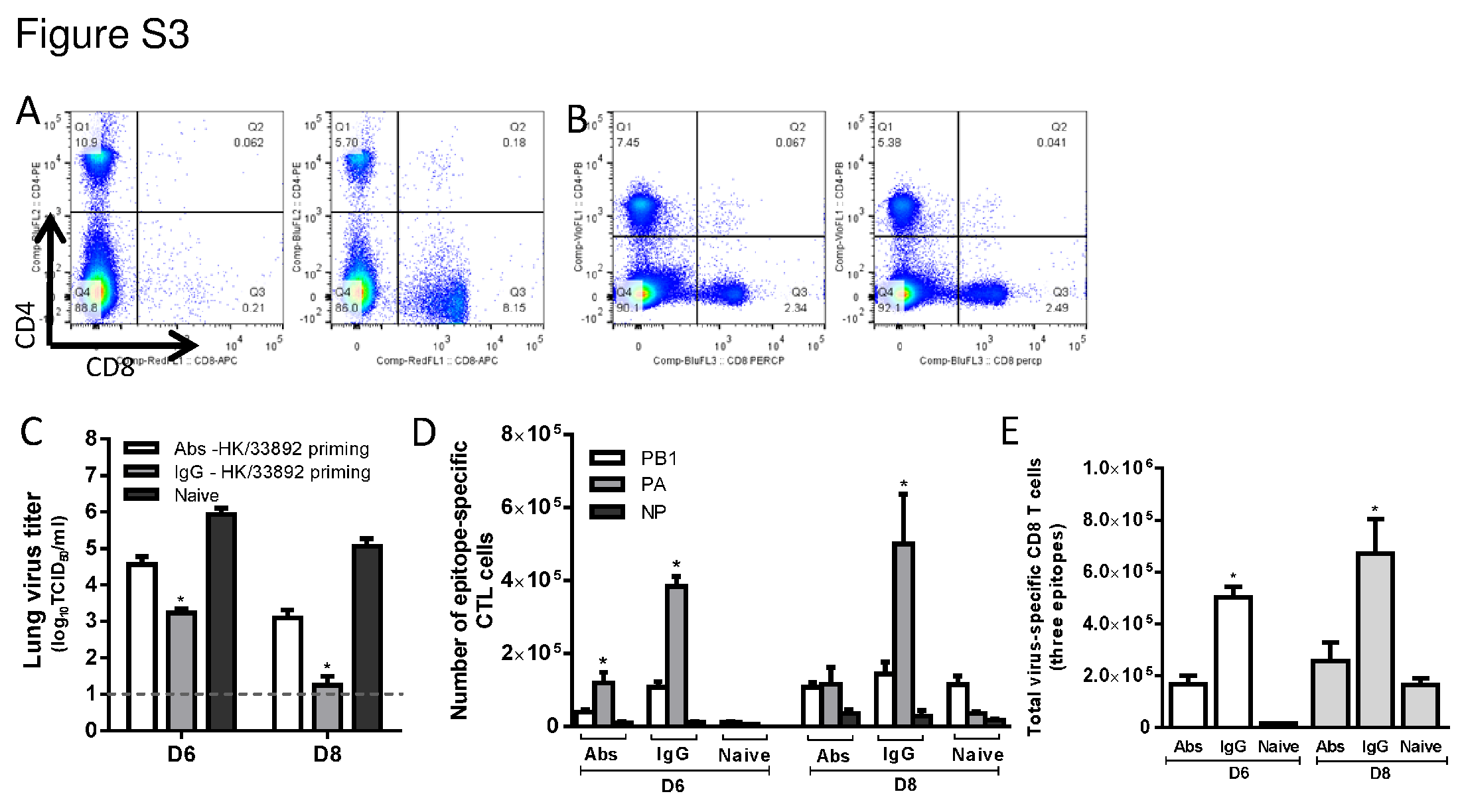

Supplement: S3 Fig — (A, B) Representative flow cytometry plots show the CD8 T cell population in blood samples after the HK33892(H9N2)-primed mice were administrated anti-CD8 antibody (Abs, left panel) or an IgG isotype control antibody (IgG, right panel) at d14 (A) and d35 p.i (B). The antibodies were injected into mice intraperitoneally at two days prior to priming infection and were further injected every three days for 2 weeks after the priming infection. They were then rested for at least a month prior to challenge with the H7N9 virus. (C)The virus titer in the lung and (D) the number of each epitope-specific CTL population (E) the combined total number of three epitope-specific CTL populations in the BALF after the antibody-treated mice were challenged with 104.5 TCID50 H7N9 virus. The data sets represent mean ± SEM, n = 4–5 per group.* p<0.05, Tukey’s test, the indicated group versus the other two groups. (TIF) [file ppat.1004642.s003.tif]

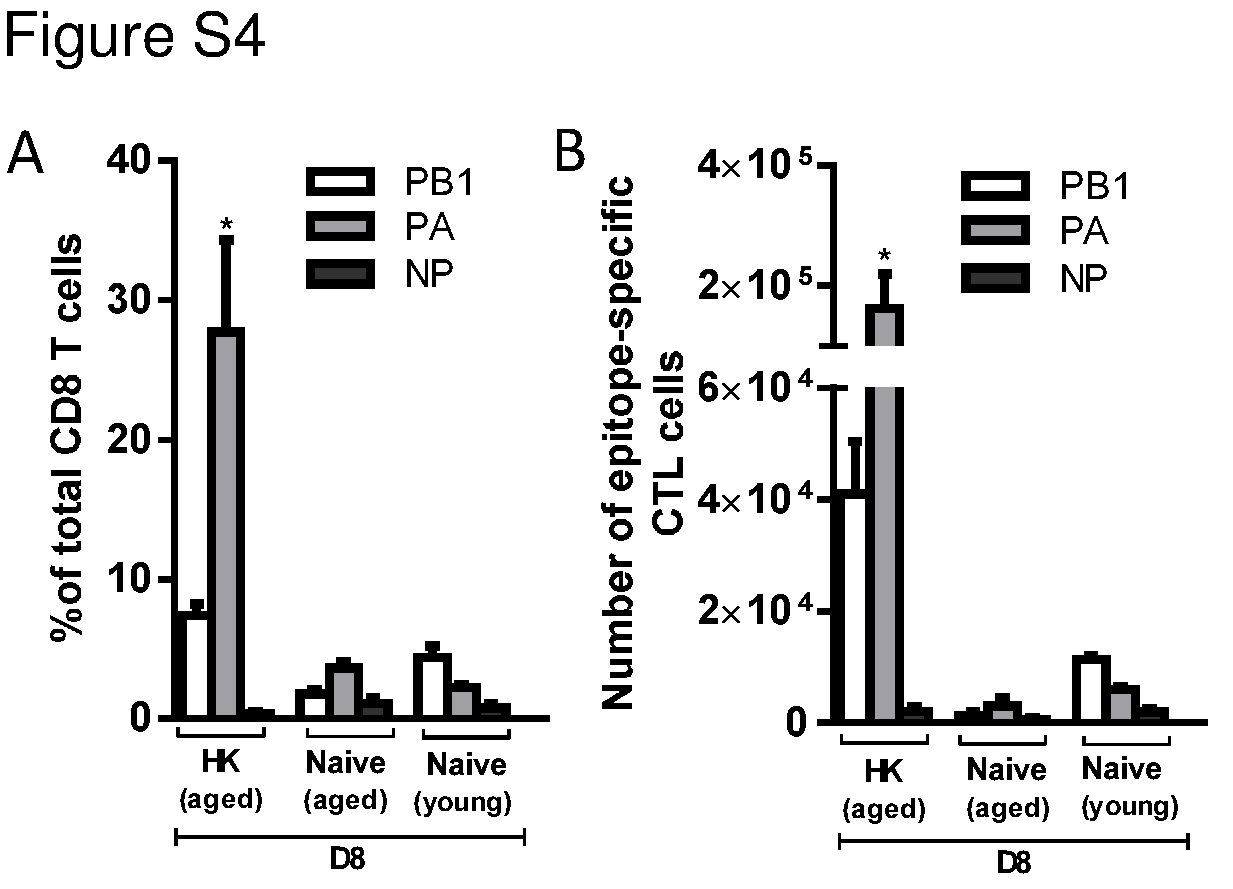

Supplement: S4 Fig — The aged female mice were between 16–18 months of age at priming and were challenged about two months after priming; age matched or young (8–10 weeks) naïve female mice were used for comparisons. The proportion (A) and number (B) of each epitope-specific CTL population in the BAL sample at d8 p.i. Data sets represent mean ± SEM, n = 3 per group. * p<0.05, Tukey’s test, the indicated epitope versus the other two epitopes. (TIF) [file ppat.1004642.s004.tif]
